# Optimizing agent behavior over long time scales by transporting value

**DOI:** 10.1038/s41467-019-13073-w

**Published:** 2019-11-19

**Authors:** Chia-Chun Hung, Timothy Lillicrap, Josh Abramson, Yan Wu, Mehdi Mirza, Federico Carnevale, Arun Ahuja, Greg Wayne

**Affiliations:** DeepMind, 5 New Street Square, London, EC4A 3TW UK

**Keywords:** Learning algorithms, Information technology

## Abstract

Humans prolifically engage in mental time travel. We dwell on past actions and experience satisfaction or regret. More than storytelling, these recollections change how we act in the future and endow us with a computationally important ability to link actions and consequences across spans of time, which helps address the problem of long-term credit assignment: the question of how to evaluate the utility of actions within a long-duration behavioral sequence. Existing approaches to credit assignment in AI cannot solve tasks with long delays between actions and consequences. Here, we introduce a paradigm where agents use recall of specific memories to credit past actions, allowing them to solve problems that are intractable for existing algorithms. This paradigm broadens the scope of problems that can be investigated in AI and offers a mechanistic account of behaviors that may inspire models in neuroscience, psychology, and behavioral economics.

## Abstract (web summary)

People are able to mentally time travel to distant memories and reflect on the consequences of those past events. Here, the authors show how a mechanism that connects learning from delayed rewards with memory retrieval can enable AI agents to discover links between past events to help decide better courses of action in the future.

## Introduction

The theory of how humans express preferences and make decisions to ensure future welfare is a question of long-standing concern, dating to the origins of utility theory^1^. Within multiple fields, including economics and psychology^2–4^, there remains unresolved debate about the appropriate formalism to explain valuation of temporally distant outcomes in long-term decision making.

In artificial intelligence (AI) research, the problem of evaluating the utility of individual actions within a long sequence is known as the credit assignment problem^5–7^. This evaluation can rate past actions or planned future actions^8^. To address credit assignment, deep learning has been combined with reinforcement learning (RL) to provide a class of architectures and algorithms that can be used to estimate the utility of courses of action for behaving, sensorimotor agents.

These algorithms have almost exclusively borrowed the assumptions of discounted utility theory^1,9,10^ and achieve credit assignment using value function bootstrapping and backpropagation (deep RL)^11^. Practical RL algorithms discount the future, reducing their applicability for problems with long delays between decisions and consequences^12,13^.

Conspicuously, humans and animals evidence behaviors that state-of-the-art (model-free) deep RL cannot yet simulate behaviorally. In particular, much behavior and learning takes place in the absence of immediate reward or direct feedback. For example, there is no standard model of effects like latent learning^14,15^, prospective memory^16^, and inter-temporal choice^2^. In sum, much human learning and decision-making occurs either without task reward or when rewards are recouped at long delay from choice points. It has been argued that hominid cognitive ability became truly modern when new strategies for long-term credit assignment (LTCA) through mental time travel and planning emerged^17^, leading to abrupt cultural shifts and immense changes in social complexity^18^. Algorithmic progress on problems of LTCA may similarly magnify the range of decision-making problems that can be addressed computationally.

Our paradigm builds on deep RL but introduces principles for credit assignment over long time scales. First, agents must encode and store perceptual and event memories; second, agents must predict future rewards by identifying and accessing memories of those past events; third, they must revaluate these past events based on their contribution to future reward.

Based on these principles, the Temporal Value Transport (TVT) algorithm uses neural network attentional memory mechanisms to credit distant past actions for later rewards. This algorithm automatically splices together temporally discontiguous events, identified by task relevance and their association to each other, allowing agents to link actions with their consequences. The algorithm is not without heuristic elements, but we prove its effectiveness for a set of tasks requiring LTCA over periods that pose enormous difficulties to deep RL.

## Results

### Episodic RL

We consider the setting of episodic RL, with time divided into separate trials (episodes) terminating after $T$ time steps. This setting is common and benefits from many practical algorithms, although other formalisms exist^19^. The agent’s behavior is governed by parameters ${\boldsymbol{\theta }}$, and it operates in the environment by receiving at each discrete time step $t$ sensory observations ${{\bf{o}}}_{t}$, processing those observations into an internal representation ${{\bf{h}}}_{t}={\bf{h}}({{\bf{o}}}_{0},\ldots ,{{\bf{o}}}_{t};{\boldsymbol{\theta }})$, and emitting actions ${{\bf{a}}}_{t}$ using a policy probability distribution $\pi ({{\bf{a}}}_{t}| {{\bf{h}}}_{t},{{\bf{y}}}_{t};{\boldsymbol{\theta }})$ (${{\bf{y}}}_{t}$ is included to allow conditioning variables). Each episode is independent of the rest save for changes due to agent learning.

The objective is to maximize the sum of rewards that the agent receives until the final time step. Let ${{\mathcal{R}}}_{t}\equiv {r}_{t}+{r}_{t+1}+\cdots +{r}_{T}$, where ${r}_{t}$ is the reward at time step $t$ and ${{\mathcal{R}}}_{t}$ is called the return. The return of any episode is non-deterministic due to randomness in the start state of the system and the random action choices of the policy. Therefore, beginning from the start of the episode the aim is to maximize the expected return, known as the value

1 $${V}_{0} = {{\mathbb{E}}}_{\pi} [{{\mathcal{R}}}_{0}] ={{\mathbb{E}}}_{\pi} \left[ \sum\limits_{t=0}^{T} {r}_{t} \right].$$

To improve performance, it is common to evaluate the episodic policy gradient^20,21^, which under fairly general conditions can be shown to have the form:

2 $$\begin{array}{ccc}{\nabla }_{{\boldsymbol{\theta }}}{V}_{0}&=&{\nabla }_{{\boldsymbol{\theta }}}{{\mathbb{E}}}_{\pi }\left[\sum\limits_{t=0}^{T}{r}_{t}\right]\\ &=&{{\mathbb{E}}}_{\pi }\left[\sum \limits_{t=0}^{T}{\nabla }_{{\boldsymbol{\theta }}}{\mathrm{log}}\pi ({{\bf{a}}}_{t}| {{\bf{h}}}_{t};{\boldsymbol{\theta }}){{\mathcal{R}}}_{t}\right]\end{array},$$

where ${\nabla }_{{\boldsymbol{\theta }}}$ is the gradient with respect to $\theta$. This quantity is typically estimated by running episodes and sampling actions from the policy probability distribution and calculating:

3 $${\nabla }_{{\boldsymbol{\theta }}}{V}_{0}\approx \Delta {\boldsymbol{\theta }}=\sum _{t=0}^{T}{\nabla }_{{\boldsymbol{\theta }}}{\mathrm{log}}\pi ({{\bf{a}}}_{t}| {{\bf{h}}}_{t};{\boldsymbol{\theta }}){{\mathcal{R}}}_{t}.$$

In practice, updating the parameters of the agent using Eq. (3) is only appropriate for the simplest of tasks, because, though its expectation is the episodic policy gradient, it is a stochastic estimate with high variance. That is, for the gradient estimate $\Delta {\boldsymbol{\theta }}$, ${\text{Var}}_{\pi }(\Delta {\boldsymbol{\theta }})$ is large relative to the magnitude of the expectation in Eq. (2). Most applications of RL mitigate this variance in two ways. First, they utilize variance reduction techniques, e.g., replacing ${{\mathcal{R}}}_{t}$ by a mean-subtracted estimate ${{\mathcal{R}}}_{t}-{\hat{V}}_{t}$, where ${\hat{V}}_{t}$ is a learned prediction of ${{\mathcal{R}}}_{t}$^10^. In this work, we use variance reduction techniques but sometimes suppress mention of them (see “Methods: Loss functions”).

Another approach to reduce variance is to introduce bias^22^ by choosing a parameter update direction $\Delta {\boldsymbol{\theta }}$ that does not satisfy ${{\mathbb{E}}}_{\pi }[\Delta {\boldsymbol{\theta }}]={\nabla }_{{\boldsymbol{\theta }}}{V}_{0}$. One of the most common tools used to manipulate bias to reduce variance is temporal discounting, which diminishes the contribution of future rewards. We define the discounted return as ${{\mathcal{R}}}_{t}^{(\gamma )}={r}_{t}+\gamma {r}_{t+1}+{\gamma }^{2}{r}_{t+2}+\cdots +{\gamma }^{T-t}{r}_{T}$. The parameter $\gamma \in [0,1]$ is known as the discount factor. For $\gamma =0.99$, a reward $100\ (=\frac{1}{1-\gamma })$ steps into the future is attenuated by a multiplicative factor of

4 $$0.9{9}^{100}={\left(1-\frac{1}{100}\right)}^{100}\approx 1/e.$$

In general, the half-life (strictly, the $1/e$-life) of reward in units of time steps is $\tau =\frac{1}{1-\gamma }$. Because effectively fewer reward terms are included in the policy gradient, the variance of the discounted policy gradient estimate

5 $${\nabla }_{{\boldsymbol{\theta }}}{V}_{0}^{(\gamma )}\approx \sum_{t=0}^{T}{\nabla }_{{\boldsymbol{\theta }}}{\mathrm{log}}\pi ({{\bf{a}}}_{t}| {{\bf{h}}}_{t};{\boldsymbol{\theta }}){{\mathcal{R}}}_{t}^{(\gamma )}$$

is smaller, but because the influence of future reward on present value is exponentially diminished, discounting limits the time scale to which an agent’s behavior is adapted to roughly a multiple of the half-life. Owing to this limitation, RL applications focus on relatively short time-scale problems, such as reactive games^11^. Yet there is a clear gap between these and relevant human time scales: much of the narrative structure of human life is characterized by highly correlated, sparse events separated by long intervals and unrelated activities.

**Fig. 1:**
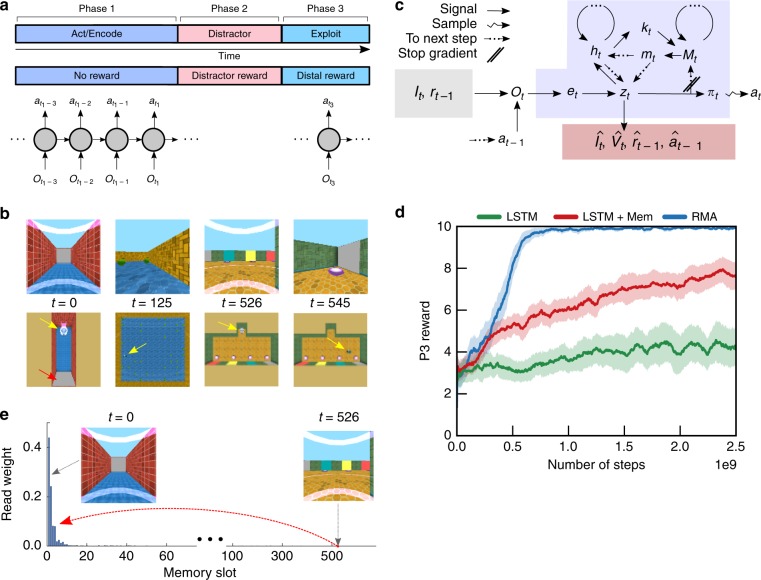
Task setting and Reconstructive Memory Agent. **a** The three-phase task structure. In phase 1 (P1), there is no reward, but the agent must seek information or trigger an event. In phase 2 (P2), the agent performs a distractor task that delivers reward. In phase 3 (P3), the agent can acquire a distal reward, depending on its behavior in P1. At each time step, the RL agent takes in observations ${{\bf{o}}}_{t}$ and produces actions ${{\bf{a}}}_{t}$ and passes memory state to the next time step. **b** The Passive Visual Match task: the agent passively observes a colored square on the wall in P1 (gray here), consumes apples in P2, and must select from a lineup of the previously observed square from P1. The agent and colored square are indicated by the yellow and red arrow, respectively. **c** The Reconstructive Memory Agent (RMA) takes in observations, ${{\bf{o}}}_{t}$, encodes them, ${{\bf{e}}}_{t}$, compresses them into a state variable ${{\bf{z}}}_{t}$, and decodes from ${{\bf{z}}}_{t}$ the observations and value prediction ${\hat{V}}_{t}$. The state variable is also passed to an RNN controller ${{\bf{h}}}_{t}$ that can retrieve (or read) memories ${t}_{t}$ from the external memory ${M}_{t}$ using content-based addressing with search keys ${{\bf{k}}}_{t}$. ${{\bf{z}}}_{t}$ is inserted into the external memory at the next time step, and the policy ${\pi }_{t}$ stochastically produces an action ${{\bf{a}}}_{t}$ as a function of $({{\bf{z}}}_{t,t},{{\bf{h}}}_{t})$ (only ${{\bf{z}}}_{t}$ shown). **d** The RMA solves the Passive Visual Match, achieving better performance than a comparable agent without the reconstruction objective (and decoders), LSTM+Mem, and better than an agent without external memory, LSTM. An agent that randomly chooses in P3 would achieve a score of $3.25$. Learning curves show standard error about the mean, computed over five independent runs. **e** The RMA uses its attentional read weight on time step 526 in P3 to retrieve the memories stored on the first few time steps in the episode in P1, when it was facing the colored square, to select the corresponding square and acquire the distal reward, worth ten points

To study decision-making in the face of long delays and intervening activity, we formalize task structures of two basic types. Each is composed of three phases (Fig. 1a), P1–P3. In the first task type (information acquisition tasks), in P1 the agent must, without immediate reward, explore an environment to acquire information; in P2, the agent engages in an unrelated distractor task over a long time period with numerous incidental rewards; in P3, the agent must exploit the information acquired in P1 to acquire a distal reward. In the second task type (causation tasks), the agent must act to trigger some event in P1 that has only long-term causal consequences. P2 is similarly a distractor task, but in P3 the agent must now exploit the changes in environment provoked by its activity in P1 to achieve success. Because a critical component of the solution we will propose involves memory encoding and retrieval, we consider P1 to consist of action followed by memory encoding, P2 as the distractor, and P3 as exploitation (Fig. 1a). While we sometimes report the performance in P2, to ensure agents perform comparably on the distractor task, we will focus primarily on the performance obtained by the agent in P3 as the quantity of interest. The challenge is to produce behavior in P1 that assists performance in P3, thereby achieving LTCA. While this task structure is contrived, it enables us to systematically control delay durations and variance in the distractor reward.

Under these assumptions, we can understand why a distractor phase can be damaging to LTCA by defining a measure of signal-to-noise ratio (SNR) in the policy gradient estimate that induces behavioral adaptation in P1. Here we measure SNR as the squared length of the expected gradient, $\parallel\!{{\mathbb{E}}}_{\pi }[\Delta {\boldsymbol{\theta }}]{\parallel }^{2}$, divided by the variance of the gradient estimate, ${\text{Var}}_{\pi }[\Delta {\boldsymbol{\theta }}]$ (the trace of ${\text{Cov}}_{\pi }(\Delta {\boldsymbol{\theta }},\Delta {\boldsymbol{\theta }})$). In Supplementary Methods 1, we show that with $\gamma =1$ the SNR is approximately

6 $${\rm{SNR}}\approx \frac{\parallel {{\mathbb{E}}}_{\pi }[\Delta {\boldsymbol{\theta }}]{\parallel }^{2}}{{\text{Var}}_{\pi }\left[\right.{\sum }_{t\in P2}{r}_{t}\left]\right.\times C({\boldsymbol{\theta }})+{\text{Var}}_{\pi }[\Delta {\boldsymbol{\theta }}| \,\text{no P2}\,]},$$

where $C({\boldsymbol{\theta }})$ is a reward-independent term and ${\text{Var}}_{\pi }[\Delta {\boldsymbol{\theta }}| \,\text{no P2}\,]$ is the (trace of the) policy gradient variance in an equivalent problem without a distractor interval. ${\text{Var}}_{\pi }\left[\right.{\sum }_{t\in P2}{r}_{t}\left]\right.$ is the reward variance in P2. When P2 reward variance is large, the policy gradient SNR is inversely proportional to it. Reduced SNR is known to adversely affect stochastic gradient optimization^23^. The standard solution is to average over larger data batches, which, with independent samples, linearly increases SNR. However, this is at the expense of data efficiency and becomes more difficult with increasing delays and interceding variance.

Before we examine a complete task of this structure, consider a simpler task, which we call Passive Visual Match (Fig. 1b), that involves a long delay and memory dependence without LTCA. It is passive in that the information that must be remembered by the agent is observed without any action required on its part; tasks of this form have been recently studied in memory-based RL^24,25^. In Passive Visual Match, the agent begins each episode in a corridor facing a wall with a painted square whose color is random. While this corresponds to the period P1 in the task structure, the agent does not need to achieve any goal. After 5 s, the agent is transported in P2 to another room where it engages in the distractor task of collecting apples for 30 s. Finally, in P3 the agent is transported to a third room in which four colored squares are posted on the wall, one of which matches the observation in P1. If the agent moves to the groundpad in front of the matching colored square, it receives a distal reward, which is much smaller than the total distractor phase reward. To solve this task, it is unnecessary for the agent to take into account reward from the distant future to make decisions as the actions in P3 precede reward by a short interval. However, the agent must store and access memories of its past to choose the pad in P3.

### The Reconstructive Memory Agent (RMA)

We solve this task with an AI agent, which we name the RMA (Fig. 1c), simplified from a previous model^24^. Critically, this model combines a reconstruction process to compress useful sensory information with memory storage that can be queried by content-based addressing^26–28^ to inform the agent’s decisions. The RMA itself does not have specialized functionality to subserve LTCA but provides a basis for the operation of the TVT algorithm, which does.

The agent compresses sensory observations into a vector, ${{\bf{z}}}_{t}$, that is both propagated to the policy to make decisions and inserted into a memory system for later retrieval, using search keys (queries) that are themselves optimized by RL. The combination of this compression process with content-based retrieval allows the RMA to make effective memory-based decisions when current sensory information is insufficient. Intuitively, remembering what previously occurred is a precondition for LTCA.

In this model, an image ${I}_{t}$, the previous reward ${r}_{t-1}$, and the previous action ${{\bf{a}}}_{t-1}$ constitute the observation ${{\bf{o}}}_{t}$ at time step $t$. These inputs are processed by encoder networks and merged into an embedding vector ${{\bf{e}}}_{t}$, which is to be combined with the output of a recurrent neural network (RNN). This RNN consists of a recurrent LSTM network ${\bf{h}}$ and a memory matrix $M$ of dimension $N\times W$, where $N$ is the number of memory slots and $W$ is the same length as a vector ${\bf{z}}$. The output of this RNN and memory system from the previous time step $t-1$ consists of the LSTM output ${{\bf{h}}}_{t-1}$ and $k$ ($=3$ here) vectors of length $W$ read from memory ${{\bf{m}}}_{t-1}\equiv ({{\bf{m}}}_{t-1}^{(1)},{{\bf{m}}}_{t-1}^{(2)},\ldots ,{{\bf{m}}}_{t-1}^{(k)})$, which we refer to as memory read vectors. Together, these outputs are combined with the embedding vector by a feedforward network into a state representation ${{\bf{z}}}_{t}=f({{\bf{e}}}_{t},{{\bf{h}}}_{t-1},{{\bf{m}}}_{t-1})$. Importantly, the state representation ${{\bf{z}}}_{t}$ has the same dimension $W$ as a memory read vector. Indeed, once produced it will be inserted into the $t$th row of memory at the next time step: ${M}_{t}[t,\cdot ]\leftarrow {{\bf{z}}}_{t}$.

Before this occurs, the RNN carries out one cycle of memory reading and computation. The state representation ${{\bf{z}}}_{t}$ is provided to the RNN, alongside the previous time step’s memory read vectors ${}_{t-1}$ to produce the next ${{\bf{h}}}_{t}$. Then the current step’s memory read vectors are produced: $k$ read keys ${{\bf{k}}}_{t}^{(1)},{{\bf{k}}}_{t}^{(2)},\ldots ,{{\bf{k}}}_{t}^{(k)}$ of dimension $W$ are produced as a function of ${{\bf{h}}}_{t}$, and each key is matched against every row $n$ using a similarity measure $S({{\bf{k}}}_{t}^{(i)},{M}_{t-1}[n,\cdot ])$. The similarities are scaled by a positive read strength parameter ${\beta }_{t}^{(i)}$ (also computed as a function of ${h}_{t}$), to which a softmax over the weighted similarities is applied. This creates an attentional read weight vector ${{\bf{w}}}_{t}^{(i)}$ with dimension $N$, which is used to construct the $i$th memory read vector ${{\bf{m}}}_{t}^{(i)}={\sum }_{n=1}^{N}{{\bf{w}}}_{t}^{(i)}[n]{M}_{t}[n,\cdot ]$.

The state representation ${{\bf{z}}}_{t}$ is also sent to decoder networks whose objective functions require them to produce reconstructions ${\hat{I}}_{t},{\hat{r}}_{t-1},{\hat{{\bf{a}}}}_{t-1}$ of the observations (the carets denote approximate quantities produced by networks) while also predicting the value function $\hat{V}({{\bf{z}}}_{t})$. This process ensures that ${{\bf{z}}}_{t}$ contains useful, compressed sensory information. Such encoder–decoder models have been explored previously in RL^24,29^. Finally, the state representation ${{\bf{z}}}_{t}$ and RNN outputs $({{\bf{h}}}_{t,t})$ are provided as input to the policy network to construct the policy distribution $\pi ({{\bf{a}}}_{t}| {{\bf{z}}}_{t},{{\bf{h}}}_{t,t})$, which is a multinomial distribution over the discrete actions here. At each time step, an action ${{\bf{a}}}_{t}$ is sampled and emitted.

When trained on Passive Visual Match, all the agents succeeded at the apple collection distractor task (Supplementary Fig. 1), although only the RMA learned to get the distal reward by selecting in P3 the square color seen in P1 (Fig. 1d). A comparison agent without an external memory (LSTM agent) achieved effectively chance performance in P3, and an agent with an external memory but no reconstruction objective decoding observation data from ${{\bf{z}}}_{t}$ (LSTM+Mem agent) also performed worse. The reconstruction process in the RMA helps to build and stabilize perceptual features in ${{\bf{z}}}_{t}$ that can later be found by memory retrieval^24^. The solution of the RMA was robust. In Supplementary Fig. 2, we demonstrate equivalent results for 0-, 15-, 30-, 45-, and 60-s distractor intervals: the number of episodes required to learn remained roughly independent of delay (Supplementary Fig. 3). In addition, for more complicated stimuli consisting of CIFAR images^30^, the RMA was able to make correct matching choices (Supplementary Fig. 4).

Despite the P2 delay, Passive Visual Match does not require LTCA. The cue in P1 is automatically observed; an agent only needs to encode and retrieve a memory to transiently move to the correct pad in P3. Consequently, an agent with a small discount factor $\gamma =0.96$ ($\tau =25$ steps at $15$ frames per second, giving a 1.67-s half-life) was able to solve the task (Supplementary Fig. 13). However, encoding and attending to specific past events was critical to the RMA’s success. In Fig. 1e, we see an attentional weighting vector ${w}_{t}$ produced by an RMA read key in an episode at time step 526, which corresponds to the beginning of P3. The weighting was focused on memories written in P1, during the instants when the agent was encoding the colored square. The learned memory retrieval identified relevant time points over the 30-s distractor interval. Recall of memories in the RMA is driven by predicting the value function $\hat{V}({{\bf{z}}}_{t})$ and producing the policy distribution $\pi ({{\bf{a}}}_{t}| {{\bf{z}}}_{t},{{\bf{h}}}_{t,t})$. As we have seen, these objectives allowed the agent to automatically detect relevant past moments.

**Fig. 2:**
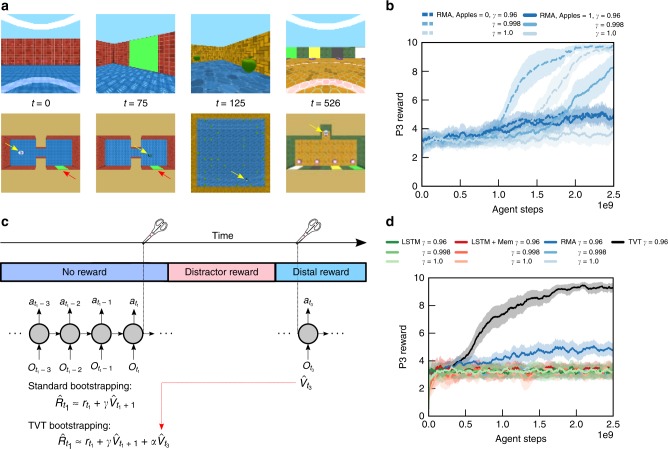
Temporal Value Transport and type 1 information acquisition tasks. **a** First person (upper row) and top–down view (lower row) in Active Visual Match task while the agent is engaged in the task. In contrast to Passive Visual Match, the agent must explore to find the colored square, randomly located in a two-room environment. The agent and colored square are indicated by the yellow and red arrow, respectively. **b** Without rewards in P2, RMA models with large discount factors (near 1) were able to solve the task; the RMA with $\gamma =0.998$ exhibited retarded but definite learning with modest P2 reward (1 point per apple). **c** Cartoon of the Temporal Value Transport mechanism: the distractor interval is spliced out, and the value prediction ${\hat{V}}_{{t}_{3}}$ from a time point ${t}_{3}$ in P3 is directly added to the reward at time ${t}_{1}$ in P1. **d** The TVT agent alone was able to solve Active Visual Match with large rewards during the P2 distractor (Supplementary Movie 1) and faster than agents exposed to no distractor reward. The RMA with discount factor $\gamma =0.96$ was able to solve a greater than chance fraction because it could randomly encounter the colored square in P1 and retrieve its memory in P3. In **b**, **d**, error bars represent standard errors across five agent training runs

We now turn to a type 1 information acquisition task, Active Visual Match, that demands LTCA. Here, in P1, the agent must actively find a colored square, randomly located in a two-room maze, so that it can decide on the match in P3 (Fig. 2a). If an agent finds the visual cue by chance in P1, then it can use this information in P3, but this will only be successful at random. As in Passive Visual Match, the agent engages in a 30-s distractor task of apple collection during P2. When the rewards of P2 apples were set to 0, RMAs with discount factors sufficiently close to 1 were able to solve the task (Fig. 2b, dashed lines). With a randomized number of apples worth one point each, the RMAs with $\gamma =0.998$ ultimately began to learn the task (Fig. 2b, solid line, medium blue) but were slower in comparison to the no P2 reward case. For a fixed mean reward per episode in P2 but increasing variance, RMA performance degraded entirely (Supplementary Fig. 5). Finally, for the principal setting of the level, where each P2 apple is worth five points and the P2 reward variance is $630$, all comparison models (LSTM agent, LSTM+Mem agent, and RMA) failed to learn P1 behavior optimized for P3 (Fig. 2d). For $\gamma =0.96$, RMAs reached a score of about 4.5, which implies slightly better than random performance in P3: RMAs solved the task in cases where they accidentally sighted the cue in P1.

### Temporal Value Transport

TVT is a learning algorithm that augments the capabilities of memory-based agents to solve LTCA problems. The insight is that we can combine attentional memory access with RL to fight variance by automatically discovering how to ignore it, effectively transforming a problem into one with no delay. A standard technique in RL is to estimate the return for the policy gradient calculation by bootstrapping^10^: using the learned value function, which is deterministic and hence low variance but biased, to reduce the variance in the return calculation. We denote this bootstrapped return as ${\tilde{R}}_{t}:= {r}_{t}+\gamma {\hat{V}}_{t+1}$. The agent with TVT (and the other agent models) likewise bootstraps from the next time step and uses a discount factor to reduce variance further. However, it additionally bootstraps from the distant future.

In Fig. 2c, we highlight the basic principle behind TVT. We previously saw in the Passive Visual Match task that the RMA reading mechanism learned to retrieve a memory from P1 in order to produce the value function prediction and policy in P3. This was an process determined automatically by the needs of the agent in P3. We exploit this phenomenon to form a link between the time point ${t}_{3}$ (occurring, e.g., in P3) at which the retrieval occurs and the time ${t}_{1}$ at which the memory was encoded. This initiates a splice event in which the bootstrapped return calculation for ${t}_{1}$ is revaluated to ${\tilde{R}}_{{t}_{1}}:= {r}_{{t}_{1}}+\gamma {\hat{V}}_{{t}_{1}+1}+\alpha {\hat{V}}_{{t}_{3}}$, where $\alpha$ is a form of discount factor that diminishes the impact of future value over multiple stages of TVT. From the perspective of learning at time ${t}_{1}$, the credit assignment is conventional: the agent tries to estimate the value function prediction based on this revaluated bootstrapped return, and it calculates the policy gradient based on it too. The bootstrapped return can trivially be regrouped, ${\tilde{R}}_{{t}_{1}}:=({r}_{{t}_{1}}+\alpha {\hat{V}}_{{t}_{3}})+\gamma {\hat{V}}_{{t}_{1}+1}$, which facilitates the interpretation of the transported value as fictitious reward introduced to time ${t}_{1}$.

This is broadly how TVT works. However, there are further practicalities. First, the TVT mechanism only triggers when a memory retrieval is sufficiently strong: this occurs whenever a read strength ${\beta }_{t}^{(i)}$ is above a threshold, ${\beta }_{\text{threshold}}$ (for robustness analyses of the reading and threshold mechanisms, see Supplementary Figs. 14, 15, 17, and 18). Second, each of the $k$ memory reading processes operates in parallel, and each can independently trigger a splice event. Third, instead of linking to a single past event, the value at the time of reading $t^{\prime}$ is transported to all times $t$ with a strength proportional to ${w}_{t^{\prime} }[t]$. Fourth, value is not transported to events that occurred very recently, where recently is any time within one half-life $\tau =1/(1-\gamma )$ of the reading time $t^{\prime}$. (See Supplementary Methods Section 2 for algorithm pseudocode.)

**Fig. 3:**
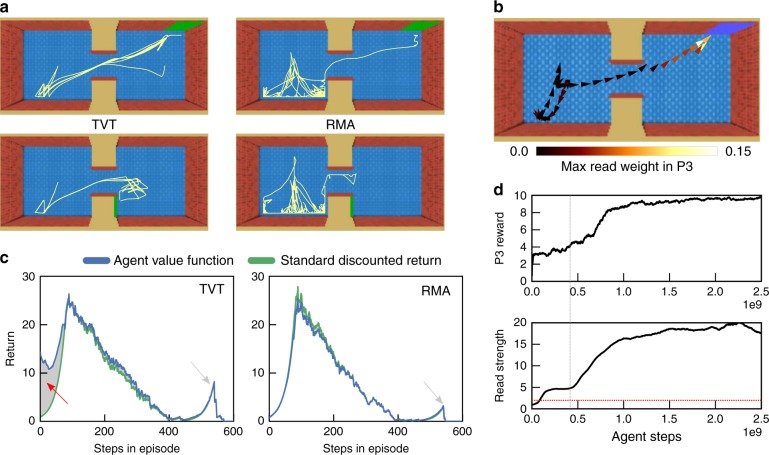
Analysis of agent in Active Visual Match. **a** In P1, TVT trained on Active Visual Match actively sought out and oriented to the colored squared. RMA meandered randomly. **b** Its attentional read weights focused maximally on the memories from time points when it was facing the colored square. **c** With statistics gathered over 20 episodes, TVT’s average value function prediction in P1 (blue) was larger than the actual discounted reward trace (green)—due to the transported reward. Difference shown in gray. The RMA value function in contrast matched the discounted return very closely. **d** The P3 rewards for TVT rose during learning (upper panel) after the maximum read strength per episode first crossed threshold on average (lower panel, red line)

When applied to the Active Visual Match task with large distractor reward, an RMA model with TVT (henceforth TVT) learned the correct behavior in P1 and faster even than RMA with no distractor reward (Fig. 2b, d). The difference in learned behavior was dramatic: TVT reliably sought out the colored square in P1, while RMA behaved randomly (Fig. 3a). Figure 3b overlays on the agent’s trajectory (arrowheads) a coloring based on the read weight produced at the time ${t}_{3}$ of a TVT splice event in P3: TVT read effectively from memories in P1 encoded while viewing the colored square. During the learning process, we see that the maximum read strength per episode (Fig. 3d, lower panel) began to reach threshold (lower panel, red line) early and prior to producing P3 reward reliably (Fig. 3d, upper panel), which instigated learning in P1. After training, TVT’s value function prediction ${\hat{V}}_{t}$ directly reflected the fictitious rewards. Averaged over 20 trials, the value function in P1 (Fig. 3c, left panel, blue curve) was higher than the actual discounted return, ${\sum }_{t^{\prime} \ge t}{\gamma }^{t^{\prime} -t}{r}_{t^{\prime} }$, (Fig. 3c, left panel, green curve). The RMA with discounting did not show a similar difference between the discounted return and the value function (Fig. 3c, right panel). In both Fig. 3c panels, we see bumps in P3 in the return traces due to the distal reward: TVT achieved higher reward in general, with the RMA return reflecting chance performance. Further, we showed TVT could solve problems with even longer distractor intervals, in this case with a P2 interval of 60 s (Supplementary Fig. 6).

**Fig. 4:**
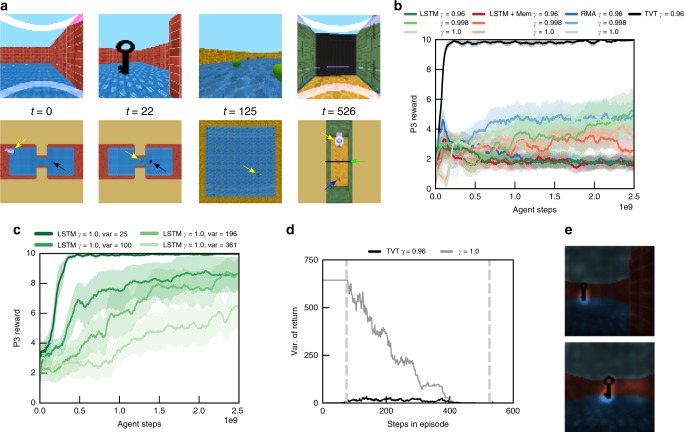
Type 2 causation tasks. **a** First person (upper row) and top–down view (lower row) in Key-to-Door task. The agent (indicated by yellow arrow) must pick up a key in P1 (black arrow), collect apples in P2, and, if it possesses the key, it can open the door (green arrow) in P3 to acquire the distal reward (blue arrow) (Supplementary Movie 2). **b** Learning curves for P3 reward (TVT in black). Although this task requires no memory for the policy in P3, computing the value prediction still triggers TVT splice events, which promote key retrieval in P1. **c** Increasing the variance of reward available in P2 disrupted the performance of LSTM agents at acquiring the distal reward. **d** On 20 trials produced by a TVT agent, we compared the variance of the TVT bootstrapped return against the undiscounted return. The TVT return’s variance was orders of magnitude lower. Vertical lines mark phase boundaries. **e** Saliency analysis of the pixels in the input image in P1 that the value function gradient is sensitive to. The key pops out in P1. In **b**, **c**, error bars represent standard errors across five agent training runs

TVT can also solve type 2 causation tasks, where the agent does not need to acquire information in P1 for P3 but instead must cause an event that will affect the state of the environment in P3. Here we study the Key-to-Door (KtD) task in which an agent must learn to pick up a key in P1 so that it can unlock a door in P3 to obtain reward (Fig. 4a). Although no information from P1 must be recalled in P3 to inform the policy’s actions (the optimal decision is to move toward the door in P3 regardless of the events in P1), TVT still learned to acquire the key in P1 because it read from memory to predict the value function when positioned in front of the door in P3 (Fig. 4b, black). All other agents failed to pick up the key reliably in P1 (Fig. 4b blue, pink, green). We parametrically changed the variance of reward in P2 (Fig. 4c and Supplementary Fig. 11). In cases where the P2 reward variance was low (SNR high), even LSTM agents with $\gamma =1$ were able to solve the task, indicating that a large memory was not the primary factor in success. However, LSTM agents could learn only for small values of P2 reward variance; performance degraded with increasing variance (Fig. 4c, dark to light green curves). In type 2 causation tasks, the TVT algorithm specifically assisted credit assignment in low SNR conditions. For the same setting as Fig. 4b, we calculated the variance of the TVT bootstrapped return ${\tilde{R}}_{t}$ for each time point, over 20 episodes, and compared on the same episodes to the variance of the undiscounted return, ${\sum }_{t^{\prime} \ge t}{r}_{t^{\prime} }$ (Fig. 4d). Because it exploits discounting, the variance of the bootstrapped return of TVT was nearly two orders of magnitude smaller in P1. We next asked whether the agent attributed the fictitious reward transported to P1 in an intelligent way to the key pickup. In P1, using a saliency analysis^31^, we calculated the derivative of the value prediction with respect to the image ${\nabla }_{{I}_{t}}{\hat{V}}_{t}({z}_{t})$ and shaded the original input image proportionally to its magnitude (Supplementary Methods 7). In Fig. 4e, we see this produced a direct segmentation of the key. As a control experiment, in Supplementary Fig. 7, we tested whether there needed to be any surface similarity between visual features in P3 and the encoded memory in P1. With a blue instead of black key, TVT also solved the task as easily, indicating that the memory searches could flexibly find information with a somewhat arbitrary relationship to current context.

The introduction of transported value can come at a cost. When a task has no need for LTCA, spurious triggering of splice events can send value back to earlier time points and bias behavior. To study this issue, we examined a set of independent tasks designed for standard discounted RL. We compared the performance of the LSTM agent, the LSTM+Mem agent, RMA, and TVT. TVT generally performed on par with RMA on many tasks but slightly worse on one, Arbitrary Visuomotor Mapping (AVM) (Supplementary Figs. 8 and 9), and outperformed all of the other agent models, including LSTM+Mem. In AVM, memory access is useful but LTCA unnecessary.

TVT could also function when P3 reward was strictly negative, but action in P1 could avert disaster. In the Two Negative Keys task (Supplementary Fig. 10), the agent is presented with a blue key and red key in a room in P1. If the agent picks up the red key, it will be able to retrieve a P3 reward behind a door worth $-1$; if it picks up the blue key, it will be able to retrieve a reward worth $-10$, and if it does not pick up a key at all, it is penalized $-20$ in P3.

Having established that TVT was able to solve simple problems, we now demonstrate TVT’s capability in two more complex scenarios. The first of these is an amalgam of the KtD and the Active Visual Match task, which demonstrates TVT across multiple phases—the Key-to-Door-to-Match task (KtDtM); here an agent must exhibit two non-contiguous behaviors to acquire distal reward.

**Fig. 5:**
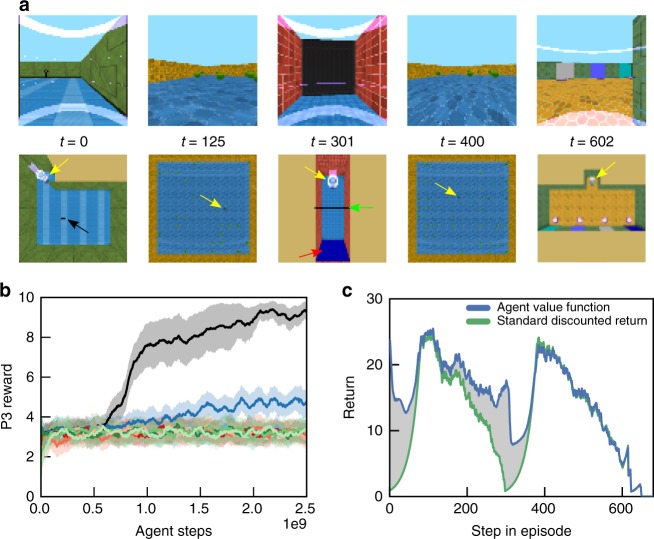
Transport across multiple phases. **a** Key-to-Door-to-Match (KtDtM) task. The agent (yellow arrow) must pick up a key (black arrow) in P1 to open a door (green arrow) and encode a colored square (red arrow) in P3 to select the matching colored square in P5 (Supplementary Movie 3). P2 and P4 are distractor apple collecting tasks. **b** TVT (black) solved this task, whereas RMA (blue) solved the P5 component of the task when it by chance retrieved the P1 key and opened the door in P3. **c** The value function prediction (blue) in TVT developed two humps where it was above the discounted return trace (green), one in P1, one in P3, encoding the value of achieving the “sub-goals” in P1 and P3

In this task, we have phases P1–P5 (Fig. 5a). P2 and P4 are both long distractor phases involving apple collection distractor rewards. In P1 and P3, there are no rewards. In P1, the agent must fetch a key, which it will use in P3 to open a door to see a colored square. In P5, the agent must choose the groundpad in front of the colored square matching the one behind the door in P3. If the agent does not pick up the key in P1, it is locked out of the room in P3 and cannot make the correct P5 choice. TVT solved this task reliably (Fig. 5b), whereas all other agents solved this problem only at chance in P5 and did not pursue the key in P1. As might be expected, the TVT value function prediction rose in P1, P3, and P5 (Fig. 5c) with two humps where the P1 and P3 value functions were above the discounted return traces. Because the discount factor $\alpha$ for TVT transport was relatively large (0.9), the two humps in the value prediction were of comparable magnitude.

**Fig. 6:**
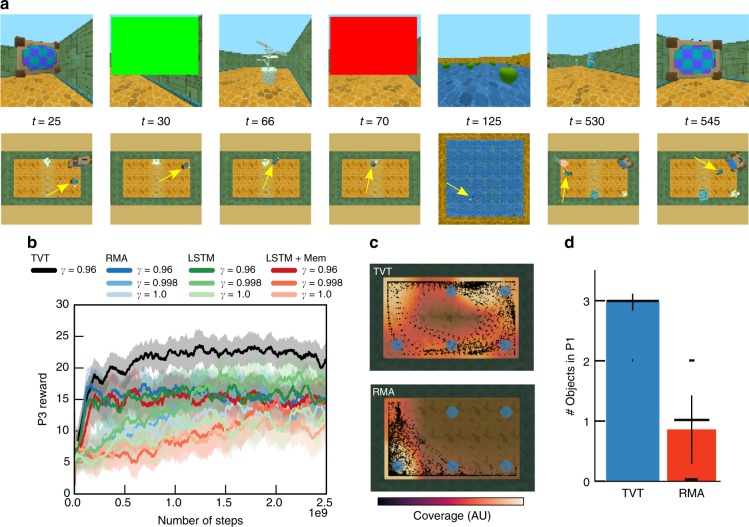
More complex information acquisition. **a** In Latent Information Acquisition, the agent (yellow arrow) must touch three procedurally generated objects to identify from a subsequent color flash if each is either green or red. In P3, green objects yield positive reward and red objects negative. **b** TVT performed well on this task (black curve; Supplementary Movie 4). The non-TVT agents touched all objects in P3 without regard to their value, thus achieving on average 15 points, whereas TVT discriminated between the good and bad objects. Error bars represent standard errors across five agent training runs. **c** In 20 trials, we plot the positional coverage in P1 of a TVT agent compared to RMA. TVT developed exploratory behavior in P1: it navigated among the six possible locations where the P1 objects could be placed, whereas the RMA typically moved into the corner. **d** A quantification over 50 trials of the exploratory behavior in P1: TVT usually touched all three of the objects in P1, whereas RMA touched about one. Each dot represents the value in one trial. The error bars indicate one standard deviation

Finally, we look at a richer task, Latent Information Acquisition (Fig. 6a). In P1, the agent begins in a room surrounded by three objects with random textures and colors drawn from a set. During P1, each object has no reward associated with it. When an object is touched by the agent, it disappears and a color swatch (green or red) appears on the screen. Green swatches indicate that the object is good and red swatches bad. The number of green- and red-associated objects was balanced. In P2, the agent again collects apples for 30 s. In P3, the agent must collect only the objects associated with green.

The TVT agent alone solved the task (Fig. 6b, black curve), usually touching all three objects in P1 (Fig. 6d), while the RMA only touched one object on average (Fig. 6b, other colors). In P1, the objects were situated on a grid of six possible locations (with no relationship to P3 location). Only TVT learned an exploratory sweeping behavior whereby it efficiently covered the locations where the objects were present (Fig. 6c); RMA reliably moved into the same corner, thus touching by accident one object.

## Discussion

There is abundant literature on interactions between memory and RL in both neuroscience and AI. Research has particularly stressed the capacity for episodic memory to support rapid learning by quick revision of knowledge regarding the structure of the environment^24,25,32–34^. By contrast, comparatively little attention has been accorded to how episodic memory can support LTCA in AI.

The mechanism of TVT should be compared to other recent proposals to address the problem of LTCA. The SAB algorithm^35^ in a supervised learning context uses attentional mechanisms over the states of an RNN to backpropagate gradients effectively. The idea of using attention to the past is shared; however, there are substantial differences. Instead of propagating gradients to shape network representations, in the RMA we have used reconstruction objectives to ensure that relevant information is encoded. Further, backpropagating gradients to RNN states would not actually train a policy’s action distribution, which is the crux of RL. Our approach instead modifies the rewards from which the full policy gradient is derived. Like TVT, RUDDER^36^ has recently been proposed in the RL context to address the problem of learning from delayed rewards. RUDDER uses an LSTM to make predictions about future returns and sensitivity analysis to distribute those returns as rewards throughout the episode. TVT explicitly uses a reconstructive memory to compress high-dimensional observations in partially observed environments and retrieve them with content-based attention. At present, we know of no other algorithm that can solve type 1 information acquisition tasks.

TVT is a heuristic algorithm and one we expect will be improved upon. In tasks where only short-term credit assignment is needed, transporting value biases the policy objective and can be counter-productive (Supplementary Figs. 8 and 9). It is possible that new methods can be developed that exhibit no bias and that are almost always helpful. Further, although TVT improved performance on problems requiring exploration, for the game Montezuma’s Revenge, which requires the chance discovery of an elaborate action sequence to observe reward, the TVT mechanism was not triggered (Supplementary Fig. 21; see Supplementary Fig. 20 for an Atari game where it did improve results).

However, TVT expresses coherent principles we believe will endure: past events are encoded, retrieved, and revaluated. TVT fundamentally connects memory and RL: attention weights on memories specifically modulate the reward credited to past events. While not intended as a neurobiological model, there is much evidence supporting the notion that short-term credit assignment and LTCA is dependent on episodic memory^34^. Numerous studies of hippocampal lesions to rats have demonstrated increases in impulsivity and discounting^4^. Further, there is evidence consistent with the theme that episodic memory^37^, planning, attention, and credit assignment are inter-related and underlie decision-making with delayed reward^38,39^. In one study, human subjects cued to recall planned future events were willing to trade immediate monetary rewards for larger monetary rewards contemporaneous to those events; quantitatively, the subjects discounted the future less than control subjects^4^. In a study of action sequence learning, subjects were found to master early actions in the sequence first; however, in an attentionally disrupted condition, the subjects mastered the later actions first—those nearer in time to reward. Here explicit attention was a necessary component of non-local temporal credit assignment^40^, a feature of TVT.

Throughout this work, we have seen that RL algorithms are compromised when solving even simple tasks requiring long-term behavior. We view discounted utility theory, upon which most RL is predicated, as the source of the problem, and our work provides evidence that other paradigms are not only possible but can work better. In economics, paradoxical violation of discounted utility theory has led to diverse, incompatible, and incomplete theories^2^. We hope that a cognitive mechanisms approach to understanding inter-temporal choice—where choice preferences are decoupled from a rigid discounting model—will inspire ways forward. The principle of linking remote events based on episodic memory access may offer a promising vantage for future study.

The complete explanation of how we problem solve and express coherent behaviors over long spans of time remains a profound mystery about which our work only provides insight. TVT learns slowly, whereas humans can quickly discover causal connections over long intervals. Human cognition is conjectured to be more model based than current AI models^41^ and can provide causal explanations^42^. When the book is written, it will likely be understood that LTCA recruits nearly the entirety of our cognitive apparatus, including systems designed for prospective planning, abstract reasoning, commitment to goals over indefinite intervals, and language. Some of this human ability may well require explanation on a different level of inquiry altogether: among different societies, norms regarding savings and investment vary enormously^43^. There is in truth no upper limit to the time horizons we can contemplate.

## Methods

### Agent model

At a high level, the RMA consists of four modules: an encoder for processing observations at each time step; a memory augmented RNN, which contains a deep LSTM controller network and an external memory that stores a history of the past; its output combines with the encoded observation to produce a state variable representing information about the environment (state variables also constitute the information stored in memory); a policy that takes the state variable and the memory’s recurrent states as input to generate an action distribution; a decoder, which takes in the state variable and predicts the value function as well as all current observations.

We now describe the model in detail by defining its parts and the loss functions used to optimize it. Parameters given per task are defined in Supplementary Table 1.

The encoder is composed of three sub-networks: the image encoder, the action encoder, and the reward encoder. These act independently on the different elements contained within the input set ${{\bf{o}}}_{t}\equiv ({I}_{t},{{\bf{a}}}_{t-1},{r}_{t-1})$, where ${I}_{t}$ is the current observed image and ${{\bf{a}}}_{t-1}$ and ${r}_{t-1}$ are the action and reward of previous time step. The outputs from these sub-networks are concatenated into a flat vector ${{\bf{e}}}_{t}$.

The image encoder takes in image tensors ${I}_{t}$ of size $64\times 64\times 3$ (3 channel RGB). We then apply six ResNet^44^ blocks with rectified linear activation functions. All blocks have 64 output channels and bottleneck channel sizes of 32. The strides for the 6 blocks are $(2,1,2,1,2,1)$, resulting in 8-fold spatial down-sampling of the original image. Therefore, the ResNet module outputs tensors of size $8\times 8\times 64$. We do not use batch normalization^45^, a pre-activation function on inputs, or a final activation function on the outputs. Finally, the output of the ResNet is flattened (into a $4096$-element vector) and then propagated through one final linear layer that reduces the size to 500 dimensions, upon which a $\tanh$ nonlinearity is applied.

In all environments, the action from the previous time step is a one-hot binary vector ${{\bf{a}}}_{t-1}$ (6-dimensional here) with ${{\bf{a}}}_{0}\equiv 0$. We use an identity encoder for the action one-hot. The reward from the previous time step ${r}_{t-1}$ is also processed by an identity encoder.

The decoder is composed of four sub-networks. Three of these sub-networks are matched to the encoder sub-networks of image, previous action, and previous reward. The fourth sub-network decodes the value function. The image decoder has the same architecture as the encoder except the operations are reversed. In particular, all two-dimensional (2D) convolutional layers are replaced with transposed convolutions^46^. In addition, the last layer produces a number of output channels that parameterize the likelihood function used for the image reconstruction loss, described in more detail in Eq. (8). The reward and action decoders are both linear layers from the state variable, ${{\bf{z}}}_{t}$, to, respectively, a scalar dimension and the action cardinality.

The value function predictor is a multi-layer perceptron (MLP) that takes in the concatenation of the state variable with the action distribution’s logits, where, to ensure that the value function predictor learning does not modify the policy, we block the gradient (stop gradient) back through to the policy logits. The MLP has a single hidden layer of $200$ hidden units and a $\tanh$ activation function, which then projects via another linear layer to an one-dimensional output. This function is a state-value function ${\hat{V}}_{t}^{\pi }\equiv {\hat{V}}^{\pi }({{\bf{z}}}_{t},\ {\rm{StopGradient}}(\mathrm{log}{\pi }_{t}))$.

The RNN is primarily based on a simplification of the Differentiable Neural Computer (DNC)^24,28^. It is composed of a deep LSTM and a slot-based external memory. The LSTM has recurrent state $({{\bf{h}}}_{t},{{\bf{c}}}_{t})$ (output state and cells, respectively). The memory itself is a 2D matrix ${M}_{t}$ of size $N\times W$, where $W$ is the same size as a state variable ${\bf{z}}$ and $N$ is the number of memory slots, which is typically set to be the number of time steps in the episode. The memory at the beginning of each episode is initialized blank, namely, ${M}_{0}=0$. We also carry the memory readouts ${{\bf{m}}}_{t}\equiv [{{\bf{m}}}_{t}^{(1)},{{\bf{m}}}_{t}^{(2)},\ldots ,{{\bf{m}}}_{t}^{(k)}]$, which is a list of $k$ vectors read from the memory ${M}_{t}$, as recurrent state.

At each time step, the following steps are taken sequentially: (1) Generate the state variable ${{\bf{z}}}_{t}$ with ${{\bf{e}}}_{t}$, ${{\bf{h}}}_{t-1}$, and ${{\bf{m}}}_{t-1}$ as input; (2) Update the deep LSTM state with ${{\bf{h}}}_{t}=\,\text{LSTM}\,({{\bf{z}}}_{t},{{\bf{m}}}_{t-1},{{\bf{h}}}_{t-1})$; (3) Construct the read key and read from the external memory; (4) Write the state variable ${{\bf{z}}}_{t}$ to slot $t$ in the external memory.

#### State variable generation

The first step is to generate a state variable, $${{\bf{z}}}_{t}$$, combining both the new observation with the recurrent information. We take the encoded current observation $${{\bf{e}}}_{t}$$ concatenated with the recurrent information $${{\bf{h}}}_{t-1}$$ and $${{\bf{m}}}_{t-1}$$ as input through a single hidden-layer MLP with the hidden layer of size $$2\times W$$$$\tanh$$ units and output layer of size $$W$$.

#### Deep LSTMs

We use a deep LSTM^47^ of two hidden layers. Although the deep LSTM model has been described before, we describe it here for completeness. Denote the input to the network at time step $t$ as ${{\bf{x}}}_{t}$. Within a layer $l$, there is a recurrent state ${{\bf{h}}}_{t}^{l}$ and a “cell” state ${{\bf{c}}}_{t}^{l}$, which are updated based on the following recursion (with $\sigma (x)\equiv {(1+\exp (-x))}^{-1}$):

$$\begin{array}{l}{{\bf{gi}}}_{t}^{l}=\sigma \left({W}_{i}^{l}[{{\bf{x}}}_{t},{{\bf{h}}}_{t-1}^{l},{{\bf{h}}}_{t}^{l-1}]+{{\bf{b}}}_{i}^{l}\right)\\ {{\bf{gf}}}_{t}^{l}=\sigma \left({W}_{f}^{l}[{{\bf{x}}}_{t},{{\bf{h}}}_{t-1}^{l},{{\bf{h}}}_{t}^{l-1}]+{{\bf{b}}}_{f}^{l}\right)\\ {{\bf{c}}}_{t}^{l}={{\bf{gf}}}_{t}^{l}\odot {{\bf{c}}}_{t-1}^{l}+{{\bf{gi}}}_{t}^{l}\odot \tanh \left({W}_{c}^{l}[{{\bf{x}}}_{t},{{\bf{h}}}_{t-1}^{l},{{\bf{h}}}_{t}^{l-1}]+{{\bf{b}}}_{c}^{l}\right)\\ {{\bf{go}}}_{t}^{l}=\sigma \left({W}_{o}^{l}[{{\bf{x}}}_{t},{{\bf{h}}}_{t-1}^{l},{{\bf{h}}}_{t}^{l-1}]+{{\bf{b}}}_{o}^{l}\right)\\ {{\bf{h}}}_{t}^{l}={{\bf{go}}}_{t}^{l}\tanh ({{\bf{c}}}_{t}^{l}),\end{array}$$

where $\odot$ is an element-wise product. To produce a complete output ${{\bf{h}}}_{t}$, we concatenate the output vectors from each layer: ${{\bf{h}}}_{t}\equiv [{{\bf{h}}}_{t}^{1},{{\bf{h}}}_{t}^{2}]$. These are passed out for downstream processing.

#### LSTM update

At each time step $t$, the deep LSTM receives input ${{\bf{z}}}_{t}$, which is then concatenated with the memory readouts at the previous time step ${{\bf{m}}}_{t-1}$. The input to the LSTM is therefore ${{\bf{x}}}_{t}=[{{\bf{z}}}_{t},{{\bf{m}}}_{t-1}]$. The deep LSTM equations are applied, and the output ${{\bf{h}}}_{t}$ is produced.

#### External memory reading

A linear layer is applied to the LSTM’s output ${{\bf{h}}}_{t}$ to construct a memory interface vector ${{\bf{i}}}_{t}$ of dimension $k\times (W+1)$. The vector ${{\bf{i}}}_{t}$ is then segmented into $k$ read keys ${{\bf{k}}}_{t}^{(1)},{{\bf{k}}}_{t}^{(2)},\ldots ,{{\bf{k}}}_{t}^{(k)}$ of length $W$ and $k$ scalars ${sc}_{t}^{(1)},\ldots ,{sc}_{t}^{(k)}$, which are passed through the function ${\text{SoftPlus}}\,(x)={\mathrm{log}}(1+\exp (x))$ to produce the scalars ${\beta }_{t}^{(1)},{\beta }_{t}^{(2)}\ldots ,{\beta }_{t}^{(k)}$.

Memory reading is executed before memory writing. Reading is content based. Reading proceeds by computing the cosine similarity between each read key ${{\bf{k}}}_{t}^{(i)}$ and each memory row $j$: ${c}_{t}^{(ij)}=\cos ({{\bf{k}}}_{t}^{(i)},{M}_{t-1}[j,\cdot ])=\frac{{{\bf{k}}}_{t}^{(i)}\cdot {M}_{t-1}[j,\cdot ]}{| {{\bf{k}}}_{t}^{(i)}| | {M}_{t-1}[j,\cdot ]| }$. We then find indices ${j}_{1}^{(i)},\ldots ,{j}_{{\text{top}}_{K}}^{(i)}$ corresponding to the ${\text{top}}_{K}$ largest values of ${c}_{t}^{(ij)}$ (over index $j$). Note that, since unwritten rows of ${M}_{t-1}$ are equal to the zero vector, some of the chosen ${j}_{1},\ldots ,{j}_{{\text{top}}_{K}}$ may correspond to rows of ${M}_{t-1}$ that are equal to the zero vector.

A weighting vector of length $N$ is then computed by setting:

$${{\bf{w}}}_{t}^{(i)}[j]=\left\{\begin{array}{c}\frac{\exp ({\beta }_{t}^{(i)}{c}_{t}^{(ij)})}{\sum _{j^{\prime} \in \{{j}_{1}^{(i)},\ldots ,{j}_{{\text{top}}_{K}}^{(i)}\}}\exp ({\beta }_{t}^{(i)}{c}_{t}^{(ij^{\prime} )})},\ \,{\text{for}}\,j\in \left\{\right.{j}_{1}^{(i)},\ldots ,{j}_{{\text{top}}_{K}}^{(i)}\left\}\right.\\ {\hskip -125pt}0,\ {\hskip -5pt}\,\text{otherwise}\,.\end{array}\right.$$

Reading is restricted to slots that have been written to so far, so it is possible to use a pre-allocated memory that is larger than the number of time steps in the episode. We demonstrate this in Supplementary Fig. 16. For each key, the readout from memory is ${{\bf{m}}}_{t}^{(i)}={M}_{t-1}^{\top }{{\bf{w}}}_{t}^{(i)}$. The full memory readout is the concatenation across all read heads: ${{\bf{m}}}_{t}\equiv [{{\bf{m}}}_{t}^{(1)},\ldots ,{{\bf{m}}}_{t}^{(k)}]$.

#### External memory writing

Writing to memory occurs after reading, which we also define using weighting vectors. The write weighting ${{\bf{v}}}_{t}^{{\rm{wr}}}$ has length $N$ and always appends information to the $t$th row of the memory matrix at time $t$, i.e., ${{\bf{v}}}_{t}^{{\rm{wr}}}[i]={\delta }_{i,t}$ (using the Kronecker delta). The information we write to the memory is the state variable ${{\bf{z}}}_{t}$. Thus the memory update can be written as

7 $${M}_{t}={M}_{t-1}+{{\bf{v}}}_{t}^{{\rm{wr}}}{{\bf{z}}}_{t}^{\top },$$

#### Policy

The policy module receives ${{\bf{z}}}_{t}$, ${{\bf{h}}}_{t}$, and ${{\bf{m}}}_{t}$ as inputs. The inputs are passed through a single hidden-layer MLP with 200 $\tanh$ units. This then projects to the logits of a multinomial softmax with the dimensionality of the action space. The action ${{\bf{a}}}_{t}$ is sampled and executed in the environment.

#### Loss functions

We combine a policy gradient loss with reconstruction objectives for decoding observations. We also have a specific loss that regularizes the use of memory for TVT.

The reconstruction loss is the negative conditional log-likelihood of the observations and return, i.e., $-{\mathrm{log}}\,p({{\mathbf{o}}}_{t},{R}_{t}| {{\bf{z}}}_{t})$, which is factorized into independent loss terms associated with each decoder sub-network and is conditioned on the state variable ${{\bf{z}}}_{t}$. We use a multinomial softmax cross-entropy loss for the action, mean-squared error (Gaussian with fixed variance of 1) losses for the reward and the value function, and a Bernoulli cross-entropy loss for each pixel channel of the image. Thus we have a negative conditional log-likelihood loss contribution at each time step of

8 $$-{\mathrm{log}}\,p({{\bf{o}}}_{t},{R}_{t}| {{\bf{z}}}_{t})\equiv {\alpha }_{\text{image}}{{\mathcal{L}}}_{\text{image}}+{\alpha }_{\text{value}}{{\mathcal{L}}}_{\text{value}}+{\alpha }_{\text{rew}}{{\mathcal{L}}}_{\text{rew}}+{\alpha }_{\text{act}}{{\mathcal{L}}}_{\text{act}},$$

where

$$\begin{array}{rcl}{{\mathcal{L}}}_{\text{image}}=\sum \limits_{w=1,h=1,c=1}^{| W| ,| H| ,| C| }\left[\right.{I}_{t}[w,h,c]{\mathrm{log}}{\hat{I}}_{t}[w,h,c]+(1-{I}_{t}[w,h,c]){\mathrm{log}}(1-{\hat{I}}_{t}[w,h,c])\left]\right.,\\ {{\mathcal{L}}}_{\text{value}}=\frac{1}{2}\left[\right.| | {R}_{t}-{\hat{V}}^{\pi }({{\bf{z}}}_{t},{\rm{StopGradient}}(\mathrm{log}{\pi }_{t}))| {| }^{2}\left]\right.,\\ {{\mathcal{L}}}_{\text{rew}}=\frac{1}{2}| | {r}_{t-1}-{\hat{r}}_{t-1}| {| }^{2},\\ {{\mathcal{L}}}_{\text{act}}=\sum \limits_{i=1}^{| A| }\left[\right.{{\bf{a}}}_{t-1}[i]{\mathrm{log}}({\hat{{\bf{a}}}}_{t-1}[i])+(1-{{\bf{a}}}_{t-1}[i]){\mathrm{log}}(1-{\hat{{\bf{a}}}}_{t-1}[i])\left]\right..\end{array}$$

On all but the standard RL control experiment tasks, we constructed the target return value as ${R}_{t}={r}_{t}+\gamma {r}_{t+1}+{\gamma }^{2}{r}_{t+2}+\cdots +{\gamma }^{T-t}{r}_{T}$. For the standard RL control experiment tasks with episodes of length $T$, we use “truncation windows”^48^ in which the time axis is subdivided into segments of length ${\tau }_{\text{window}}$. We can consider full gradient as a truncated gradient with ${\tau }_{\text{window}}=T$. If the window around time index $t$ ends at time index $k$, the return within the window is

9 $${R}_{t}:=\left\{\begin{array}{cc} {r}_{t}+\gamma {r}_{t+1}+{\gamma }^{2}{r}_{t+2}+\cdots +{\gamma}^{k-t+1}{\hat{V}}^{\pi}({\mathbf{z}}_{k+1},{\mathrm{log}}{\pi }_{k+1}),& {\text{if}}\,k \,< \, T,\\ {\hskip -70pt}{r}_{t}+\gamma {r}_{t+1}+{\gamma }^{2}{r}_{t+2}+\cdots +{\gamma }^{T-t}{r}_{T}, & {\text{if}}\,T\le k.\end{array}\right.$$

As a measure to balance the magnitude of the gradients from different reconstruction losses, the image reconstruction loss is divided by the number of pixel channels $|W|\, \times | H| \times | C|$.

We use discount and bootstrapping parameters $\gamma$ and $\lambda$, respectively, as part of the policy advantage calculation given by the Generalized Advantage Estimation (GAE) algorithm^49^. Defining ${\delta }_{t}\equiv {r}_{t}+\gamma {\hat{V}}^{\pi }({{\bf{z}}}_{t+1},{\mathrm{log}}{\pi }_{t+1})-{\hat{V}}^{\pi }({{\bf{z}}}_{t},{\mathrm{log}}{\pi }_{t})$, GAE makes an update of the form:

10 $$\Delta \theta \propto \sum_{t=k{\tau }_{\text{window}}}^{(k+1){\tau }_{\text{window}}}\sum _{t^{\prime} =t}^{(k+1){\tau }_{\text{window}}}{(\gamma \lambda )}^{t^{\prime} -t}{\delta }_{t^{\prime} }{\nabla }_{\theta }\mathrm{log}{\pi }_{\theta }({{\bf{a}}}_{t}| {{\bf{h}}}_{t}).$$

There is an additional loss term that increases the entropy of the policy’s action distribution. This and pseudocode for all of RMA’s updates are provided in Supplementary Note 1.

For TVT, we include an additional regularization term ${{\mathcal{L}}}_{\text{read-regularization}}$.

### Comparison models

We introduce two comparison models: the LSTM+Mem Agent and the LSTM Agent. The LSTM+Mem Agent is similar to the RMA. The key difference is that it has no reconstruction decoders and losses. The value function is produced by a one hidden-layer MLP with 200 hidden units: $\hat{V}({{\bf{z}}}_{t},{\rm{StopGradient}}(\mathrm{log}{\pi }_{t}))$.

The LSTM Agent additionally has no external memory system and is essentially the same design as the A3C agent^48^. We have retrofitted the model to share the same encoder networks as the RMA, acting on input observations to produce the same vector ${e}_{t}$. This is then passed as input to a deep two-layer LSTM that is the same as the one in RMA. The LSTM has two output “heads”, which are both one hidden-layer MLPs with 200 hidden units: one for the policy distribution $\pi ({{\bf{a}}}_{t}| {{\bf{z}}}_{t},{{\bf{h}}}_{t})$ and one for the value function prediction $\hat{V}({{\bf{z}}}_{t},{{\bf{h}}}_{t},{\rm{StopGradient}}(\mathrm{log}{\pi }_{t}))$. As for our other agents, the policy head is trained using Eq. (10).

We hyper-parameter searched for the best learning rates on comparison models (Supplementary Fig. 12). The throughput in environment steps taken per second for RMA was about 70% of the LSTM agent (Supplementary Fig. 19). TVT ran as fast as RMA.

### Implementation and optimization

For optimization, we used truncated backpropagation through time^50^. We ran 384 parallel worker threads that each ran an episode on an environment and calculated gradients for learning. Each gradient was calculated after one truncation window, ${\tau }_{\text{window}}$. For all main paper experiments other than the standard RL control experiments, ${\tau }_{\text{window}}=T$, the length of the episode.

The gradient computed by each worker was sent to a “parameter server” that asynchronously ran an optimization step with each incoming gradient. We optimize the model using ADAM optimizers^51^ with ${\beta }_{1}=0.9$ and ${\beta }_{2}=0.999$.

The pseudocode for each RMA worker is presented in Supplementary Methods 2. For all experiments, we used the open source package Sonnet available at https://github.com/deepmind/sonnet. All network parameters were initialized to its parameter defaults.

### Temporal Value Transport

TVT works in two stages. First, we identify significant memory read events, which become splice events. Second, we transport the value predictions made at those read events back to the time points being read from, where they modify the rewards and therefore the RL updates.

At time $t^{\prime}$, the read strengths ${\beta }_{t^{\prime} }^{(i)}$ are calculated as described in “External Memory Reading.” To exclude sending back value to events in the near past, for time points $t^{\prime}$ where $t^{\prime} -\arg {\max }_{t}{{\bf{w}}}_{t^{\prime} }[t]\, < \, {1}/(1-\gamma )$, we reset ${\beta }_{t^{\prime} }^{(i)}:=0$ for the remainder of the computation. We then identify splice events by first finding all time windows $[t^{\prime}_- ,t^{\prime}_+]$ where ${\beta }_{t^{\prime} }^{(i)}\ge {\beta }_{\text{threshold}}$ for $t^{\prime} \in [t^{\prime}_- ,t^{\prime}_+]$ but ${\beta }_{t^{\prime} }^{(i)}\, <\,{\beta }_{\text{threshold}}$ for $t^{\prime} =t^{\prime}_- -1$ and $t^{\prime} =t^{\prime}_+ +1$.

We then set ${t}_{\max }$ to be the $\arg \max$ over $t^{\prime}$ of ${\beta }_{t^{\prime} }^{(i)}$ in the period for the included points. For each ${t}_{\max }$ above, we modify the reward of all time points $t$ occurred more than $1/(1-\gamma )$ steps beforehand:

11 $${r}_{t}\to \left\{\begin{array}{l}{r}_{t}+\alpha {{\bf{w}}}_{{t}_{\max }}^{(i)}[t]{\hat{V}}_{{t}_{\max }+1},\ \,{\text{if}}\,t \, > \, {t}_{\max }-1/(1-\gamma ),\\ {\hskip -120pt} {r}_{t},\ {\hskip -1pt} \,\text{otherwise.}\,\\ \end{array}\right.$$

We send back ${\hat{V}}_{{t}_{\max }+1}$ because that is the first value function prediction that incorporates information from the read at time ${t}_{\max }$. In addition, for multiple read processes $i$, the process is the same, with independent, additive changes to the reward at any time step. Pseudocode for TVT with multiple read processes is provided in Supplementary Methods 2.

To prevent the TVT mechanism from being triggered extraneously, we impose a small regularization cost whenever a read strength is above threshold.

12 $${{\mathcal{L}}}_{\text{read-regularization}}={\alpha }_{\text{read-regularization}}\times \sum _{i=1}^{k}\max ({\beta_{t}^{(i)}}-{\beta }_{\text{threshold}},0),$$

with ${\alpha }_{\text{read-regularization}}=5\times 1{0}^{-6}$. This is added to the other loss terms.

### Reporting summary

Further information on research design is available in the Nature Research Reporting Summary linked to this article.

## Supplementary information

Supplementary Information

Reporting Summary

Description of Additional Supplementary Files

Supplementary Movie 1

Supplementary Movie 2

Supplementary Movie 3

Supplementary Movie 4

## Data Availability

Source data for experiments is available on request. A reporting summary for this article is available as a Supplementary Information file.

## Code availability

The simulation environments and a non-distributed working agent implementation will be made available on publication at https://github.com/deepmind/tvt. Sonnet is available at https://github.com/deepmind/sonnet. DeepMind Lab is available at https://github.com/deepmind/lab.
