# Supplementary material for: Optimizing agent behavior over long time scales by transporting value
Source: Nat Commun. 2019 Nov 19;10:5223. doi: 10.1038/s41467-019-13073-w (PMC6864102; doi:10.1038/s41467-019-13073-w)
Supplement: Supplementary file 1 — Supplementary Information [file 41467_2019_13073_MOESM1_ESM.pdf]

Optimizing Agent Behavior over Long Time Scales by Transporting Value

Hung et al.

Supplementary Information

Contents

1 Supplementary Methods: Signal-to-Noise Ratio Analysis 3

2 Supplementary Methods: Algorithm Pseudocode for RMA and TVT 5

3 Supplementary Methods: Task Definitions 5

4 Supplementary Methods: Distractor Phase Modifications 11

5 Supplementary Methods: Control Tasks 11

6 Supplementary Methods: Task Specific Parameters 12

7 Supplementary Methods: Task Analyses 12

8 Supplementary Tables 16

9 Supplementary Figures 16

1 Supplementary Methods: Signal-to-Noise Ratio Analysis

As in the article text, we refer to phases 1-3 as P1-P3. We define the signal as the squared norm of the expected policy change in P1 induced by the policy gradient. To be precise, let $\Delta\theta := \sum_{t \in P1} \nabla_\theta \log \pi(\mathbf{a}_t|\mathbf{h}_t) R_t$. Further, in the following assume that the returns are baseline-subtracted, i.e. $R_t \to R_t - \mathbb{E}_\pi[R_t]$. We define the signal as

$$\begin{aligned} \text{Signal} &:= \| \mathbb{E}_\pi[\Delta\theta] \|^2 \\ &= \left\| \mathbb{E}_\pi \left[\sum_{t \in P1} \nabla_\theta \log \pi(\mathbf{a}_t|\mathbf{h}_t) \sum_{t' \geq t} r_{t'} \right] \right\|^2 . \end{aligned}$$

We define the noise as the trace of the variance of the policy gradient

$$\begin{aligned} \text{Noise} &:= \text{Tr}\left(\text{Var}_\pi[\Delta\theta] \right) \\ &= \mathbb{E}_\pi \left[\left\| \sum_{t \in P1} \nabla_\theta \log \pi(\mathbf{a}_t|\mathbf{h}_t) R_t - \mathbb{E}_\pi[\Delta\theta] \right\|^2 \right]. \end{aligned}$$

Recall that $r_t \equiv 0$ for $t \in$ P1. Further, P1 and P2 are approximately independent as P2 is a distractor phase whose initial state is unmodified by activity in P1. The only dependence is given by the agent's internal state and parameters, but we assume for these problems it is a weak dependence, which we ignore for present calculations. In this case,

$$\begin{aligned} \mathbb{E}_\pi \left[\sum_{t \in P1} \nabla_\theta \log \pi(\mathbf{a}_t|\mathbf{h}_t) \sum_{t' \geq t} r_{t'} \right] &= \mathbb{E}_\pi \left[\sum_{t \in P1} \nabla_\theta \log \pi(\mathbf{a}_t|\mathbf{h}_t) \left[\sum_{t' \in P2} r_{t'} + \sum_{t' \in P3} r_{t'} \right] \right] \\ &\approx \mathbb{E}_\pi \left[\sum_{t \in P1} \nabla_\theta \log \pi(\mathbf{a}_t|\mathbf{h}_t) \sum_{t' \in P3} r_{t'} \right]. \qquad (1) \end{aligned}$$

Based on these considerations, the signal term is easy to calculate:

$$\begin{aligned} \text{Signal} &\approx \| \mathbb{E}_\pi[\Delta\theta | \text{no P2}] \|^2 \\ &= \left\| \mathbb{E}_\pi \left[\sum_{t \in P1} \nabla_\theta \log \pi(\mathbf{a}_t|\mathbf{h}_t) \sum_{t' \in P3} r_{t'} \right] \right\|^2 . \end{aligned}$$

Define $g_\theta := \sum_{t \in P1} \nabla_\theta \log \pi(a_t|h_t)$. With this, the noise term becomes

$$\begin{aligned}
\text{Noise} &= \mathbb{E}_\pi\left[\left\|\sum_{t \in P1} \nabla_\theta \log \pi(\mathbf{a}_t|\mathbf{h}_t) \sum_{t' \geq t} r_{t'} - \mathbb{E}_\pi[\Delta\theta]\right\|^2\right] \\
&= \mathbb{E}_\pi\left[\left\|g_\theta\left[\sum_{t' \in P2} r_{t'} + \sum_{t' \in P3} r_{t'}\right] - \mathbb{E}_\pi[\Delta\theta]\right\|^2\right] \\
&= \mathbb{E}_\pi\left[\left\|g_\theta \sum_{t' \in P2} r_{t'} + \left(g_\theta \sum_{t' \in P3} r_{t'} - \mathbb{E}_\pi[\Delta\theta]\right)\right]\right\|^2\right] \\
&\approx \mathbb{E}_\pi\left[\left\|g_\theta \sum_{t' \in P2} r_{t'}\right\|^2\right] + \mathrm{Tr}\left(\mathrm{Var}_\pi[\Delta\theta|\text{no P2}]\right),
\end{aligned}$$

where $\mathrm{Tr}\left(\mathrm{Var}_\pi[\Delta\theta|\text{no P2}]\right)$ is the variance in the policy gradient due to P1 and P3 without a P2 distractor phase. (The approximate equality represents that the memory state of the system is altered by the P2 experience, but we neglect this dependence.) We make the assumption that performance in P2 is independent of activity in P1, which is approximately the case in the distractor task we present in the main text. With this assumption, the first term above becomes

$$\begin{aligned}
\mathbb{E}_\pi\left[\left\|\sum_{t \in P1} \nabla_\theta \log \pi(\mathbf{a}_t|\mathbf{h}_t) \sum_{t' \in P2} r_{t'}\right\|^2\right] &= \mathrm{Var}_\pi\left[\sum_{t' \in P2} r_{t'}\right] \times \mathbb{E}_\pi\left[\left\|\sum_{t \in P1} \nabla_\theta \log \pi(\mathbf{a}_t|\mathbf{h}_t)\right\|^2\right] \\
&= \mathrm{Var}_\pi\left[\sum_{t' \in P2} r_{t'}\right] \times \mathbb{E}_\pi\left[\left\|\sum_{t \in P1} \nabla_\theta \log \pi(\mathbf{a}_t|\mathbf{h}_t)\right\|^2\right. \\
&\quad \left. - \left\|\underbrace{\mathbb{E}_\pi\left[\sum_{t \in P1} \nabla_\theta \log \pi(\mathbf{a}_t|\mathbf{h}_t)\right]}_{=0}\right\|^2\right] \\
&= \mathrm{Var}_\pi\left[\sum_{t' \in P2} r_{t'}\right] \times \mathrm{Tr}\left(\mathrm{Var}_\pi\left[\sum_{t \in P1} \nabla_\theta \log \pi(\mathbf{a}_t|\mathbf{h}_t)\right]\right).
\end{aligned}$$

Thus, the SNR (Signal / Noise) is approximately

$$\mathrm{SNR} \approx \frac{\left\|\mathbb{E}_\pi\left[\sum_{t \in P1} \nabla_\theta \log \pi(\mathbf{a}_t|\mathbf{h}_t) \sum_{t' \in P3} r_{t'}\right]\right\|^2}{\mathrm{Var}_\pi\left[\sum_{t' \in P2} r_{t'}\right] \times \mathrm{Tr}\left(\mathrm{Var}_\pi\left[\sum_{t \in P1} \nabla_\theta \log \pi(\mathbf{a}_t|\mathbf{h}_t)\right]\right) + \mathrm{Tr}\left(\mathrm{Var}_\pi[\Delta\theta|\text{no P2}]\right)}.$$

In the limit of large P2 reward variance, we have

$$\mathrm{SNR} \approx \frac{\left\| \mathbb{E}_{\pi}\left[\sum_{t \in P1} \nabla_{\theta} \log \pi(\mathbf{a}_t | \mathbf{h}_t) \sum_{t' \in P3} r_{t'} \right] \right\|^2}{\mathrm{Var}_{\pi}\left[\sum_{t' \in P2} r_{t'} \right] \times \mathrm{Tr}\left(\mathrm{Var}_{\pi}\left[\sum_{t \in P1} \nabla_{\theta} \log \pi(\mathbf{a}_t | \mathbf{h}_t) \right] \right)}.$$

The reward variance in P2, $\mathrm{Var}_{\pi}\big[\sum_{t' \in P2} r_{t'}\big]$, reduces the policy gradient SNR, and low SNR is known to impact the convergence of stochastic gradient optimization negatively [1]. Of course, averaging S independent episodes increases the SNR correspondingly to $S \times \mathrm{SNR}$, but the approach of averaging over an increasing number of samples is not universally possible and only defers the difficulty: there is always a level of reward variance in the distractor phase that matches or overwhelms the variance reduction achieved by averaging.

2 Supplementary Methods: Algorithm Pseudocode for RMA and TVT

3 Supplementary Methods: Task Definitions

All tasks were implemented in DeepMind Lab (DM Lab) [2]. DM Lab has a standardized environment map unit length: all sizes given below are in these units. For all DM Lab experiments, agents processed 15 frames per second. The environment itself produced 60 frames per second, but we propagated only the first observation of each packet of four to the agents. Rewards accumulated over each packet were summed together and associated to the first, undropped frame. Similarly, the agents chose one action at the beginning of this packet of four frames: this action was applied four times in a row. We define the number of "Agent Steps" as the number of independent actions sampled by the agent: that means one agent step per packet of four frames.

We used a consistent action set for all experiments except for the Arbitrary Visuomotor Mapping task. For all other tasks, we used a set of six actions: *move forward*, *move backward*, *rotate left with rotation rate of 30* (mapping to an angular acceleration parameter in DM Lab), *rotate right with rotation rate of 30*, *move forward and turn left*, *move forward and turn right*. For the Arbitrary Visuomotor Mapping, we did not need to move relative to the screen, but we instead needed to move the viewing angle of the agent. We thus used four actions: *look up*, *look down*, *look left*, *look right* (with rotation rate parameter of 10).

DM Lab maps use *texture sets* to determine the floor and wall textures. We use a combination of four different texture sets in our tasks: *Pacman*, *Tetris*, *Tron* and *Minesweeper*. DM Lab texture sets can take on various colours but

Algorithm 1 RMA Worker Pseudocode

// Assume global shared model parameter vectors θ and counter $T := 0$
// Assume thread-specific parameter vectors θ'
// Assume discount factor $\gamma \in (0, 1]$ and bootstrapping parameter $\lambda \in [0, 1]$
Initialize thread step counter $t := 1$
repeat
  Synchronize thread-specific parameters $\theta' := \theta$
  Zero model's memory & recurrent state if new episode begins
  $t_{\text{start}} := t$
  repeat
    $\mathbf{e}_t = \text{Encode}(\mathbf{o}_t)$
    $\mathbf{z}_t = \text{StateVariableMLP}(\mathbf{e}_t, \mathbf{h}_{t-1}, \mathbf{m}_{t-1})$
    $\mathbf{h}_t, \mathbf{m}_t = \text{RNN}(\mathbf{z}_t, \mathbf{h}_{t-1}, \mathbf{m}_{t-1})$ // (Memory-augmented RNN)
    Update memory $M_t = \text{Write}(M_{t-1}, \mathbf{z}_t)$
    Policy distribution $\pi_t = \pi(\mathbf{a}_t | \mathbf{z}_t, \mathbf{h}_t, \mathbf{m}_t)$
    Sample $\mathbf{a}_t \sim \pi_t$
    $\hat{V}_t, \hat{\mathbf{o}}_t^r = \text{Decode}(\mathbf{z}_t, \text{StopGradient}(\log \pi_t))$
    Apply $\mathbf{a}_t$ to environment and receive reward r_t and observation $\mathbf{o}_{t+1}$
    $t := t + 1; T := T + 1$
  until environment termination or $t - t_{\text{start}} == \tau_{\text{window}}$
  If not terminated, run additional step to compute $\hat{V}(\mathbf{z}_{t+1}, \log \pi_{t+1})$
  and set $R_{t+1} := \hat{V}(\mathbf{z}_{t+1}, \log \pi_{t+1})$ // (but don't increment counters)
  (Optional) Apply Temporal Value Transport (Alg. 3)
  Reset performance accumulators $\mathcal{A} := 0; \mathcal{L} := 0; \mathcal{H} := 0$
  for k from t down to t_{start} **do**
    $\gamma_t := \begin{cases} 0, & \text{if } k \text{ is environment termination} \\ \gamma, & \text{otherwise} \end{cases}$
    $R_k := r_k + \gamma_t R_{k+1}$
    $\delta_k := r_k + \gamma_t \hat{V}(\mathbf{z}_{k+1}, \log \pi_{k+1}) - \hat{V}(\mathbf{z}_k, \log \pi_k)$
    $A_k := \delta_k + (\gamma\lambda) A_{k+1}$
    $\mathcal{A} := \mathcal{A} + A_k \log \pi_k[a_k]$
    $\mathcal{H} := \mathcal{H} - \alpha_{\text{entropy}} \sum_i \pi_k[i] \log \pi_k[i]$ // (Entropy loss)
    $\mathcal{L} := \mathcal{L} + \mathcal{L}_k$ (Methods Eq. 8)
  end for
  $d\theta' := \nabla_{\theta'}(\mathcal{A} + \mathcal{H} + \mathcal{L})$
  Asynchronously update via gradient ascent θ using $d\theta'$
until $T > T_{\text{max}}$

we use the default colours for each set, which are: Pacman: blue floors and red walls. Tetris: blue floor and yellow walls. Tron: yellow floor and green walls. Minesweeper: blue floor and green walls. Examples of how these sets appear can be seen in various figures in the main text.

Episodes for the tasks with delay intervals are broken up into multiple phases. Phases do not repeat within an episode. Generally, the tasks contain three

Algorithm 2 Temporal Value Transport for One Read

input: $\{r_t\}_{t\in[1,T]}$, $\{\hat{V}_t\}_{t\in[1,T]}$, read strengths $\{\beta_t\}_{t\in[1,T]}$, read weights $\{\mathbf{w}_t\}_{t\in[1,T]}$

```
splices : = []
for each crossing of read strength β_t above β_threshold do
  t_max := arg max_t{β_t | t ∈ crossing window}
  Append t_max to splices
end for
for t in 1 to T do
  for t' in splices do
    if t < t' − 1/(1 − γ) then
      r_t := r_t + α w_{t'}[t] V̂_{t'+1}
      {The read based on w_{t'} influences value prediction at next step, hence
      V̂_{t'+1}.}
    end if
  end for
end for
return {r_t}_{t∈[1,T]}
```

phases (P1-P3), with a middle phase.

We used a standardized P2 distractor phase task: the map is an 11×11 open square (Figure 1b second column). The agent spawns (appears) adjacent to the middle of one side of the square, facing the middle. An apple is randomly spawned independently at each unit of the map with probability 0.3, except for the square in which the agent spawns. Each apple gives a reward r_{apple} of 5 when collected and disappears after collection. The agent remains in this phase for 30 seconds. (This length was varied in some experiments.) The map uses the Tetris texture set unless mentioned otherwise.

In several tasks, we use *cue images* to provide visual feedback to the agent, e.g. indicating that an object has been picked up. These cue images are colored rectangles that overlay the input image, covering the majority of the top half of the image. An example of a red cue image is shown in Supplementary Figure 10a, third panel. These cues are shown for 1 second once activated, regardless of a transition to a new phase that may occur during display.

In each episode of Passive Visual Match, four distinct colors are randomly chosen from a fixed set of 16 colors. One of these is selected as the *target color* and the remaining three are *distractor colors.* Four squares are generated with these colors, each the size of one wall unit. The three phases in each episode are:

1. The map is a 1×3 corridor with a target color square covering the wall unit at one end. The agent spawns facing the square from the other end of the corridor (Figure 1b first column). There are no rewards in this phase. The agent remains in this phase for 5 seconds. The map uses the Pacman texture set.

Algorithm 3 Temporal Value Transport for Multiple Reads

input: $\{r_t\}_{t\in[1,T]}$, $\{\hat{V}_t\}_{t\in[1,T]}$, read strengths $\{\beta_t^{(i)}\}_{t\in[1,T],i\in[1,k]}$, read weights $\{w_t^{(i)}\}_{t\in[1,T],i\in[1,k]}$
for $i \in [1, k]$ **do**
  for $t' \in [1, T]$ **do**
    if $t' - \arg\max_t w_{t'}^{(i)}[t] < 1/(1-\gamma)$ **then**
      $\beta_{t'}^{(i)} := 0$
    end if
  end for
  splices := []
  for each crossing of read strength $\beta_t^{(i)}$ above $\beta_{\text{threshold}}$ **do**
    $t_{\max} := \arg\max_t\{\beta_t^{(i)} | t \in \text{crossing window}\}$
    Append $t_{\max}$ to splices
  end for
  for t in 1 to T **do**
    for t' in splices **do**
      if $t < t' - 1/(1-\gamma)$ **then**
        $r_t := r_t + \alpha \mathbf{w}_{t'}^{(i)}[t]\hat{V}_{t'+1}$
      end if
    end for
  end for
end for
return $\{r_t\}_{t\in[1,T]}$

2. The standard distractor phase described above.

3. The map is a 4×7 rectangle with the four color squares (the target color and three distractor colors) on one of the longer sides, with a unit gap between each square. The ordering of the four colors is randomly chosen. There is an additional single unit square placed in the middle of the opposite side, in which the agent spawns, facing the color squares. In front of each color square is a groundpad (Figure 1b last two columns). When the agent touches one of these pads, a reward of 10 points is given if it is the pad in front of the target painting and a reward of 1 is given for any other pad. The episode then ends. If the agent does not touch a pad within 5 seconds then no reward is given for this phase and the episode ends. The map uses the Tron texture set.

Active Visual Match is the same as Passive Visual Match, except that the map in phase 1 is now larger and the position of the target image in phase 1 is randomized. The phase 1 map consists of two 3×3 open squares connected by a 1×1 corridor that joins each square in the middle of one side (Figure 2a first two columns). The agent spawns in the center of one of the two squares, facing the middle of one the walls adjacent to the wall with the opening to the

corridor. The target color square is placed randomly over one of any of the wall units on the map.

The three phases of Key-to-Door are:

1. The map is identical to the map in phase 1 of Active Visual Match. The agent spawns in the corner of one the squares that is furthest from the opening to the corridor, facing into the square but not towards the opening. A key is placed randomly within the map (not at the spawn point) and if the agent touches the key it disappears and a black cue image is shown (Figure 4a first two columns). As in the Visual Match tasks, there are no rewards in this phase, and the phase lasts for 5 seconds. The map uses the Pacman texture set.

2. The standard distractor phase.

3. The map is a 1×3 corridor with a locked door in the middle of the corridor. The agent spawns at one end of the corridor, facing the door. At the end of the corridor on the other side of the door is a goal object (Figure 4a fourth column). If the agent touched the key in phase one, the door can be opened by walking into it, and then if the agent walks into the goal object a reward of 10 points is given. Otherwise, no reward is given. The map uses the Tron texture set.

Key-to-Door-to-Match combines elements of Key-to-Door with Active Visual Match. One target color and three distractor colors are chosen in the same way as for the Visual Match tasks. In contrast to the standard task setup, there are five phases per episode:

1. This phase is the same as phase 1 of Key-to-Door but with a different map. The map is a 3×4 open rectangle with an additional 1×1 square attached at one corner, with the opening on the longer of the two walls. The agent spawns in the additional 1×1 square, facing into the rectangle (Figure 5a first column). The map uses the Minesweeper texture set.

2. The standard distractor phase except that the phase lasts for only 15 seconds instead of 30 seconds.

3. The map is the same as in phase 3 of Key-to-Door. Instead of a goal object behind the locked door, the target color square covers the wall at the far end of the corridor (Figure 5a third column). There is no reward in this phase, and it lasts for 5 seconds. The map uses the Pacman texture set.

4. The standard distractor phase except that the phase lasts for only 15 seconds instead of 30 seconds.

5. The final phase is the same as phase 3 in the Visual Match tasks.

The three phases of Two Negative Keys are:

1. The map is a 3×4 open rectangle. The agent spawns in the middle of one of the shorter walls, facing into the rectangle. One red key is placed in a corner opposite the agent, and one blue key is placed in the other corner opposite the agent. Which corner has the red key and which the blue key is randomized per episode. If the agent touches either of the keys, a red or blue cue image is shown according to which key the agent touched (Supplementary Figure 10 first three columns). After one key is touched, it disappears, and nothing happens if the agent goes on to touch the remaining key (i.e., no cue is displayed and the key remains in the map). The phase lasts for 5 seconds, and there are no rewards; if the agent does not touch any key during this period, at the end of the phase a black cue image is shown. The map uses the Tron texture set.
2. The standard distractor phase except with the Tetris texture set.
3. The layout is the same as in phase 3 of the Key-to-Door task. If the agent has picked up either of the keys then the door will open when touched, and the agent can collect the goal object, at which point it will spawn back into the map from phase 2 but with all remaining apples removed. This phase lasts for only 2 seconds in total; when it ends, a reward of -20 is given if the agent did not collect the goal object; a reward of -10 is given if the agent collected the goal object after touching the blue key; and a reward of -1 is given if the agent collected the goal object after touching the red key. The map uses the Tron texture set.

In each episode of Latent Information Acquisition, three objects are randomly generated using the DM Lab object generation utilities. Color and type of object is randomized. Each object is independently randomly assigned to be a *good* or a *bad* object.

1. The map is a 3×5 rectangle. The agent spawns in one corner facing outwards along one of the shorter walls. The three objects are positioned randomly among five points as displayed in Figure 6c in the main text (Figure 6a first four columns). If an agent touches one of the good objects, it disappears, and a green cue image is shown. If an agent touches one of the bad objects, it disappears, and a red cue image is shown. This phase lasts for 5 seconds, and there are no rewards. The map uses the Tron texture set. The image cues shown in this phase are only shown for 0.25 seconds so that the cues do not interfere with continuation of the P1 activity (in all other tasks they are shown for 1 second).
2. The standard distractor phase except with the Tetris texture set.
3. The map, spawn point, and possible object locations are the same as in phase 1. The objects are the same, but their positions are randomly chosen again. If the agent touches a good object it disappears, and a reward of 20 is given. If the agent touches a bad object it disappears and a reward of -10 is given. This phase lasts for 5 seconds. The map uses the Tron texture set.

4 Supplementary Methods: Distractor Phase Modifications

In order to analyze the effect of increasing variance of distractor reward on agent learning, we created variants of the distractor phase where this reward variance could be easily controlled. Since the distractor phase is standardized, any of these modifications can be used in any of those tasks.

Zero Apple Reward: The reward given for apples in the distractor phase is zero. Even though the apples give zero reward, they still disappear when touched by the agent. Fixed Number of Apples: The reward given for apples remains at 5. Instead of the 120 free squares of the map independently spawning an apple with probability 0.3, we fix the number of apples to be $120 \times 0.3 = 36$ and distribute them randomly among the 120 available map units. Under an optimal policy where all apples are collected, this has the same mean reward as the standard distractor phase but with no variance. Variable Apple Reward: The reward r_{apple} given for apples in the distractor phase can be modified (to a positive integer value), but with probability $1 - 1/r_{\text{apple}}$ each apple independently gives zero reward instead of r_{apple}. Any apple touched by the agent still disappears.

This implies that the optimal policy and expected return under the optimal policy is constant, but variance of the returns increases with r_{apple}. Since there are 120 possible positions for apples in the distractor phase, and apples independently appear in each of these positions with probability 0.3, the variance of undiscounted returns in P2, assuming all apples are collected, is

$$120 \times \left[\left(0.3 \times \frac{1}{r_{\text{apple}}} \right) \times r_{\text{apple}}^2 - (0.3 \times 1)^2 \right] = 36 \times (r_{\text{apple}} - 0.3). \qquad (2)$$

5 Supplementary Methods: Control Tasks

Control tasks are taken from the DM Lab 30 task set [2]. The tasks we include had a memory access component to performance. We provide only brief descriptions here since these tasks are part of the open source release of DM Lab available at `https://github.com/deepmind/lab/tree/master/game_scripts/levels/contributed/dmlab30`.

In Explore Goal Locations Small, agents must find the goal object as fast as possible. Within episodes, when the goal object is found the agent respawns and the goal appears again in the same location. The goal location, level layout, and theme are randomized per episode. The agent spawn location is randomized per respawn.

In Natlab Varying Map Randomized, the agent must collect mushrooms within a naturalistic terrain environment to maximise score. The mushrooms do not regrow. The map is randomly generated and of intermediate size. The topographical variation, and number, position, orientation and sizes of shrubs, cacti and rocks are all randomized. Locations of mushrooms are randomized.

The time of day is randomized (day, dawn, night). The spawn location is randomized for each episode.

In Psychlab Arbitrary Visuomotor Mapping, a task in the Psychlab framework [3], the agent is shown images from a visual memory capacity experiment dataset [4] but in an experimental protocol known as arbitrary visuomotor mapping. The agent views consecutive images that are associated to particular cardinal directions. The agent is rewarded if it can remember the direction to move its fixation cross for each image. The images are drawn from a set of roughly 2,500 possible images, and the specific associations are randomly generated per episode.

6 Supplementary Methods: Task Specific Parameters

Across models the same parameters were used for the TVT, RMA, LSTM+Mem, and LSTM agents except for γ, which for the TVT model was always 0.96 and was varied as expressed in the figure legends for the other models. Learning rate was varied only for the learning rate analysis in Section 7.

Across tasks, we used the parameters shown in Table 1 with a few exceptions:

- For all the control tasks, we used $\alpha_{\text{image}} = 1$ instead of 20.
- For all the control tasks, we used $\tau_{\text{window}} = 200$ instead of using the full episode.
- For the Two Negative Keys task, we used $\alpha_{\text{entropy}} = 0.05$ instead of 0.01.

The TVT specific parameters were not varied across tasks. In Supplementary Figure 15, we show that $\beta_{\text{threshold}}$ could range from 1 to 5 without destroying performance on the Active Visual Match task. For the Key-to-Door task, in Supplementary Figure 14, we show that we saw no performance difference for a factor of two variation in the threshold, but, for the largest value of the threshold, performance deteriorated, as the TVT mechanism was not triggered.

7 Supplementary Methods: Task Analyses

For Active Visual Match and Key-to-Door tasks, we performed analysis of the effect of distractor phase reward variance on the performance of the agents. To do this we used the same tasks but with modified distractor phases as described in Supplementary Methods 4.

Active Visual Match

Supplementary Figure 13 shows learning curves for $r_{\text{apple}} = 0$ (zero apple reward) and $r_{\text{apple}} = 1$ (variable apple reward). When $r_{\text{apple}} = 1$, all apples give reward. Learning for the RMA was already significantly disrupted when

$r_{\text{apple}} = 1$, so for Active Visual Match we do not report higher variance examples.

Key-to-Door

Figure 4c shows learning curves with apple reward r_{apple} set to 1, 3, 6, and 10, which gives variances of total P2 reward as 25, 100, 196, and 361, respectively, (the variable apple reward condition). Note that episode scores for these tasks show that all apples are usually collected in P2 at policy convergence.

Note that the mean distractor phase return in the previous analysis is much less than the mean return in the standard distractor phase. Another way of looking at the effect of variance in the distractor phase whilst including the full mean return is shown in Supplementary Figure 11, which has three curves: one for zero apple reward, one for a fixed number of apples, and one for the full level (which has a variable number of apples per episode but the same expected return as the fixed number of apples case). From the figure, it can be seen that introducing large rewards slows learning in phase 1 due to the variance whilst the agent has to learn the policy to collect all the apples, but that the disruption to learning is much more significant when the number of apples continues to be variable even after the agent has learned the apple collection policy.

Return Prediction Saliency

To generate Figure 4e in the main text, a sequence of actions and observations for a single episode of Key-to-Door was recorded from a TVT agent trained on that level. We show two time steps where the key was visible. We calculated gradients $\partial \hat{V}_t / \partial I_t^{w,h,c}$ of the agent's value predictions with respect to the input image at each time step. We then computed the sensitivity of the value function prediction to each pixel:

$$g_t^{w,h} = \sqrt{\sum_{c=1}^{3} |\partial \hat{V}_t / \partial I_t^{w,h,c}|^2}.$$

We smoothed these sensitivity estimates using a 2 pixel-wide Gaussian filter:

$$\hat{g}_t^{w,h} = \text{GaussianFilter}(g_t^{w,h}, \sigma = 2 \text{ pixels}).$$

We then normalized this quantity based on its statistics across time and pixels by computing the 97th percentile:

$$g_{97} = \text{97th percentile of } \hat{g}_t^{w,h} \text{ over all } t, w, h.$$

Input images were then layered over a black image with an alpha channel that increased to 1 based on the sensitivity calculation. Specifically, we used an alpha

channel value of:

$$\alpha_t^{w,h} = \min\left(0.3 + (1 - 0.3)\frac{\hat{g}_t^{w,h}}{g_{97}}, 1\right). \quad (3)$$

Learning Rate Analysis for High Discount Factor

To check that the learning rates used for the high discount RMA and LSTM models were reasonable, we ran the largest variance tasks from in Section 7 (for RMA with $\gamma = 0.998$) and 7 (for LSTM with $\gamma = 0.998$) for learning rates 3.2×10^{-7}, 8×10^{-7}, 2×10^{-6}, 5×10^{-6} and 1.25×10^{-5}. The results are shown in Supplementary Figure 12, and they show that the default learning rate of 5×10^{-6} was the best among those tried.

Behavioral Analysis of Active Visual Match

We compared the P1 behaviors of a TVT agent versus an RMA as shown in Figure 3a in the main text. First, we modified the environment to fix the color square in one of three pre-selected wall locations. We then ran TVT and RMA for 10 episodes in each of these three fixed color square conditions. Finally, we plotted the agents' positional trajectories in each condition. We also visualized the TVT agent's memory retrievals by plotting a single episode trajectory with arrowheads indicating agent orientation on each second agent step. Each arrowhead is also color-coded by the maximal read weight from any time step in P3 back to the memory encoded at this time and position in P1.

Behavioral Analysis of Latent Information Acquisition

We evaluated TVT and RMA for 50 episodes in the latent information acquisition task. To visualize, we scatter-plotted the agent's position as a black dot for each P1 time step (50 episodes $\times$ 75 P1 time steps = $3,750$ dots in total). We also binned the agent's position on a 4×5 grid and counted the percentage of time the agent had occupied each grid cell. We visualized this grid occupancy using a transparent heatmap overlying the top-down view. To further quantify the behaviour of TVT versus RMA, we recorded how many objects were acquired by the agent in the exploration phase in each of the 50 test trials and plotted the mean and standard deviation in a bar plot.

Return Variance Analysis

Over 20 trials, in Key-to-Door we computed and compared two return variances based on trajectories from the same TVT agent. The first was the undiscounted return: $R_t = \sum_{t' \geq t} r_{t'}$. The second was computed as in Algorithm 1 and Algorithm 3 using TVT ($\gamma = \lambda = 0.96$), i.e., it was bootstrapped recursively:

$$\tilde{R}_t = r_t + \gamma[\lambda \tilde{R}_{t+1} + (1 - \lambda)\hat{V}_{t+1}],$$

and r_t was modified by TVT.

8 Supplementary Tables

Parameter	Value
η	5×10^{-6}
γ	various
λ	$= \gamma$
α_{image}	20
α_{rew}	1
α_{value}	0.4
α_{act}	1
α_{entropy}	0.01
τ_{window}	Number of steps in episode
N	Number of steps in episode
W	200
k	3
top_K	50
$\alpha_{\text{read-regularization}}$	5×10^{-6}
$\beta_{\text{threshold}}$	2

Supplementary Table 1: Parameters used across tasks (not all parameters apply to all models). The TVT specific parameters $\beta_{\text{threshold}}, \alpha_{\text{read-regularization}}$ were held constant across all tasks.

9 Supplementary Figures

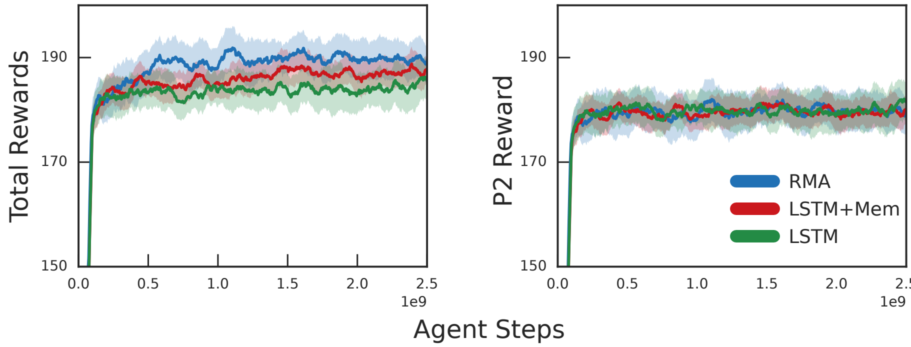

Supplementary Figure 1: **Passive Image Match Learning.** *Left.* Full episode score. *Right.* P2 score. ($\gamma = 0.96$ for all models.)

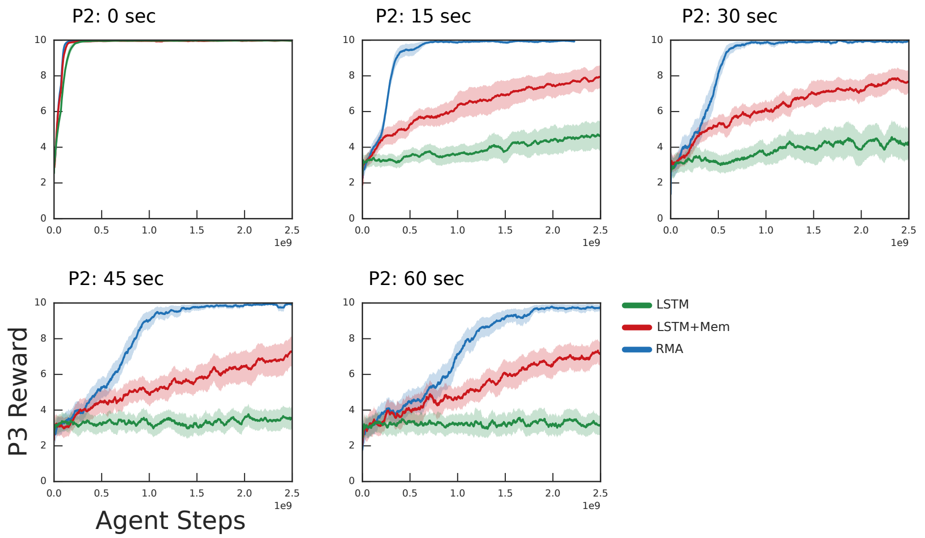

Supplementary Figure 2: **Passive Image Match with Varying Delay Period.** All models learned to retrieve the P3 reward with no P2 delay, but performance is hampered for longer delays for models with no reconstructive loss.

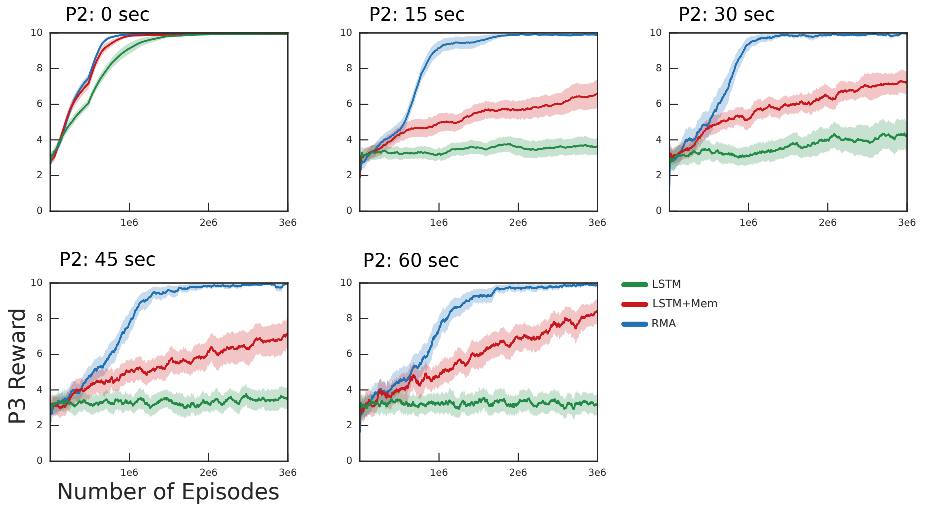

Supplementary Figure 3: **Passive Image Match with Varying Delay Period (Episodes).** With the x-axis plotted in episodes, controlling for the number of additional steps due to the delay period, the RMA learned in roughly the same number of episodes, regardless of delay length (0 seconds to 60 seconds).

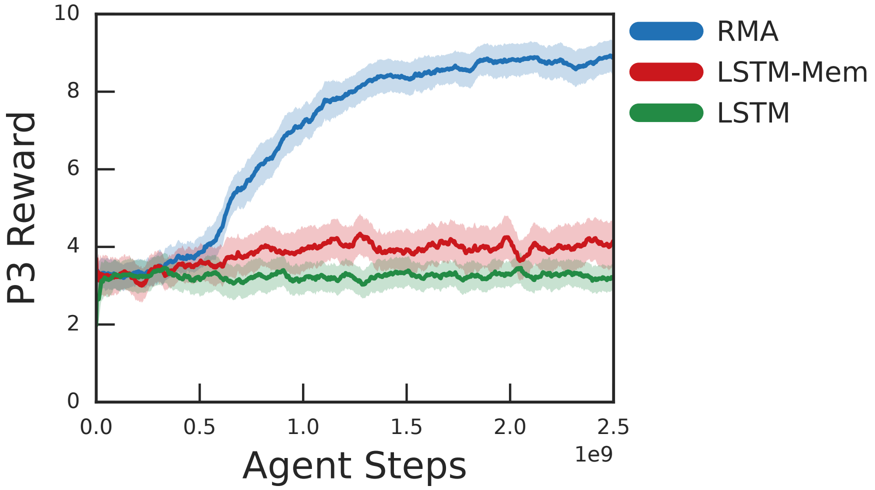

Supplementary Figure 4: **Passive Image Match (CIFAR-10).** Using CIFAR-10 images [5] instead of colored squares as P1 and P3 images, the RMA was still able to perform the Passive Image Match Task.

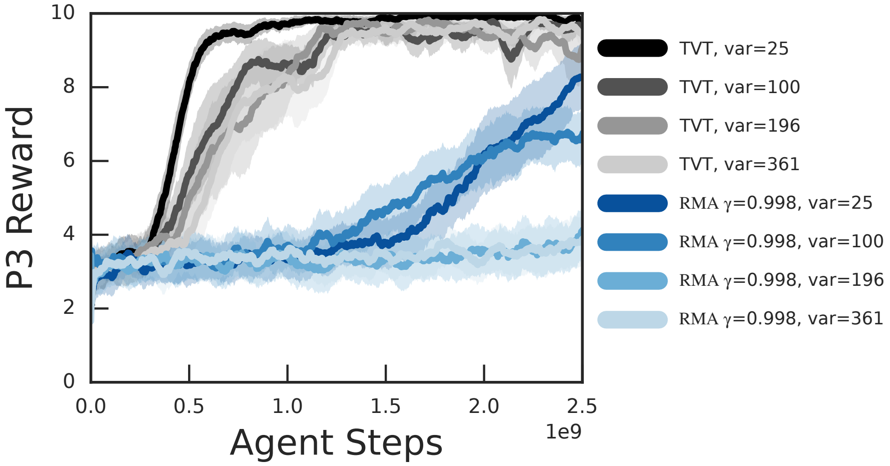

Supplementary Figure 5: **Effect of P2 Reward Variance in Active Visual Match.** P2 reward variance was introduced by varying the probability and reward value of apple reward (variable apple reward condition). For higher levels of P2 reward variance, the RMA models failed to solve Active Visual Match, though TVT was largely unaffected.

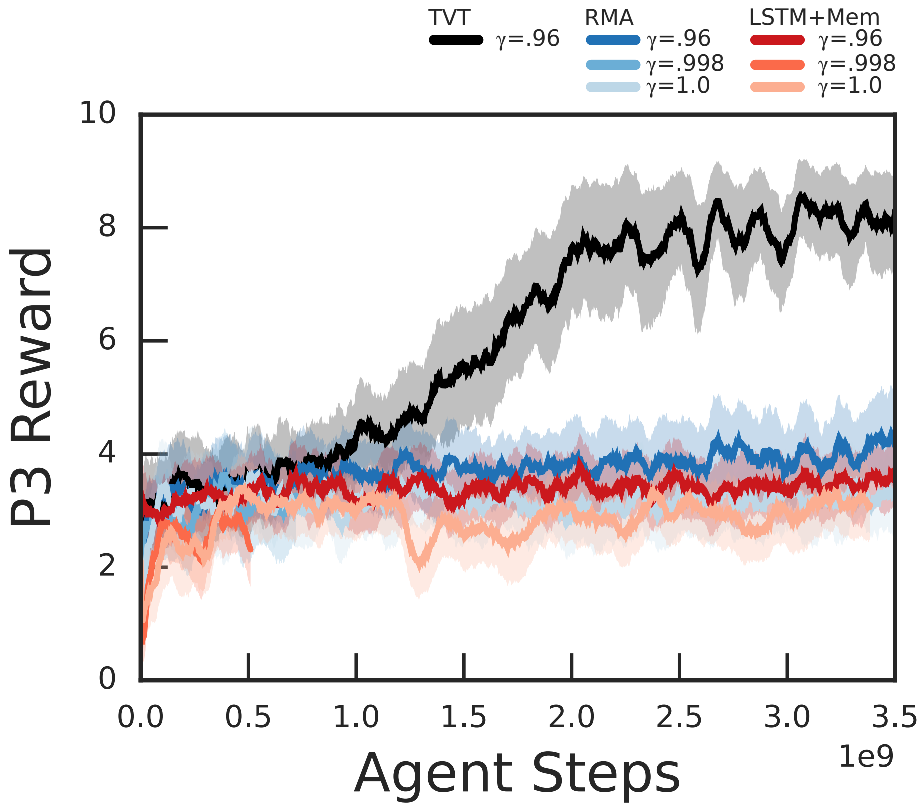

Supplementary Figure 6: **Active Visual Match 60 Second P2.** The TVT agent was also able to solve an Active Visual Match task with a 60 second P2 delay period.

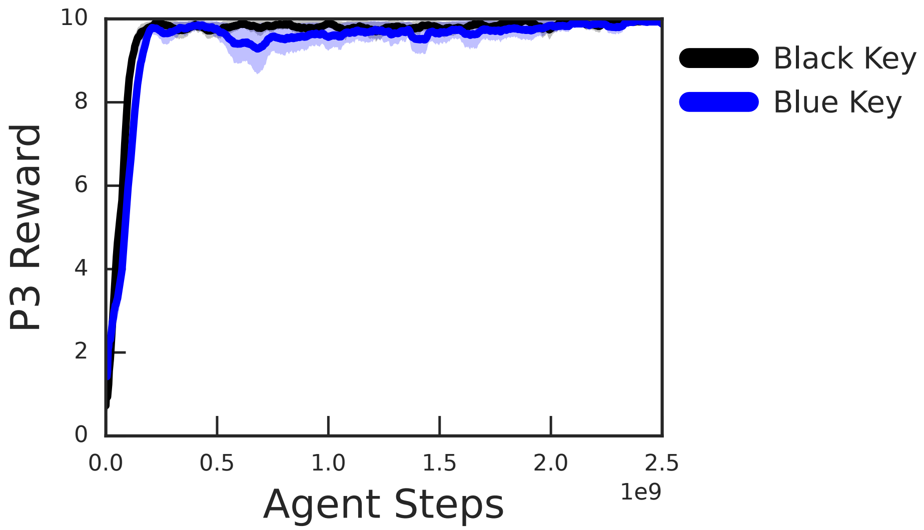

Supplementary Figure 7: **Key-to-Door: Black vs. Blue key.** With a black door in P3, TVT was able to solve the task as easily with a blue key in P1, implying that content-based memory retrieval was flexible and not based on surface similarity between the key and door color.

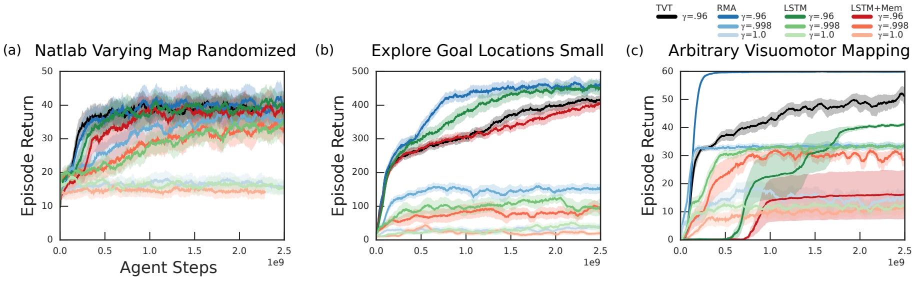

Supplementary Figure 8: **Control Task DM Lab Learning.** *a.* TVT (black) learned Natlab Varying Map Randomized just as well as the RMA. *b.* On Explore Goal Locations Small, TVT led to a modest decrement in final performance. *c.* On Psychlab Arbitrary Visuomotor Mapping, TVT did decrement final performance and slowed learning, though the agent's performance was still high compared to all but the RMA.

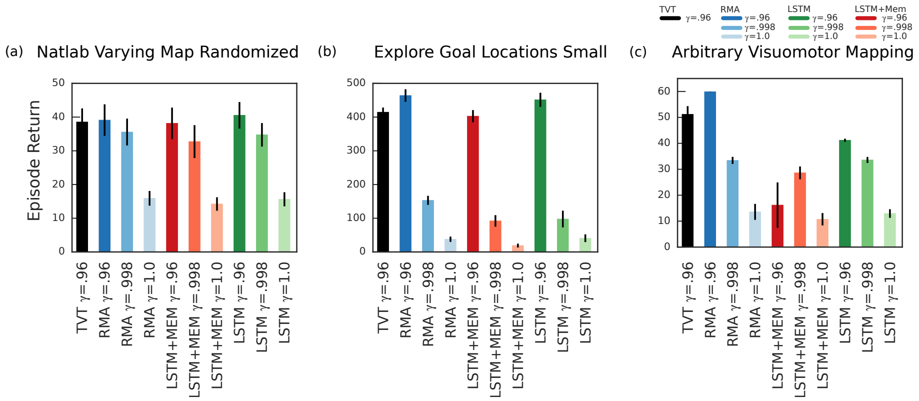

Supplementary Figure 9: **Control Task DM Lab Final Performance.** Final performance for 5 training runs from Supplementary Figure 8.

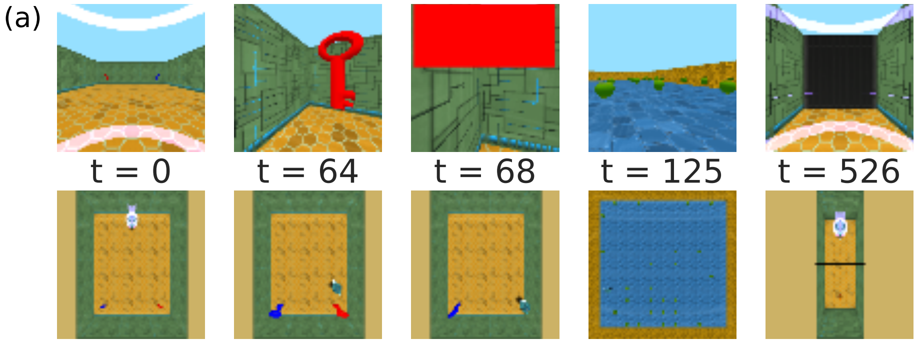

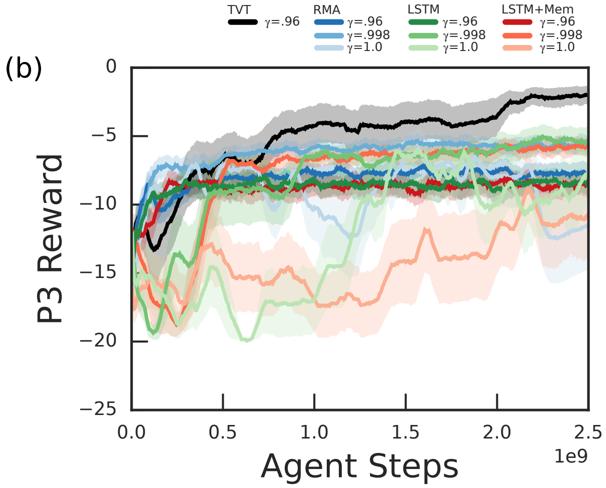

Supplementary Figure 10: **Two Negative Keys level.** *a.* In P1, the agent selects between a red and a blue key, distributed randomly in the room corners. The red key allows the agent to open the door in P3, receiving negative reward of -1. The blue key leads to negative reward of -10. No key selection leads to a negative reward of -20. *b.* TVT was able to solve this task, picking up the red key, and receiving -1 on average in P3.

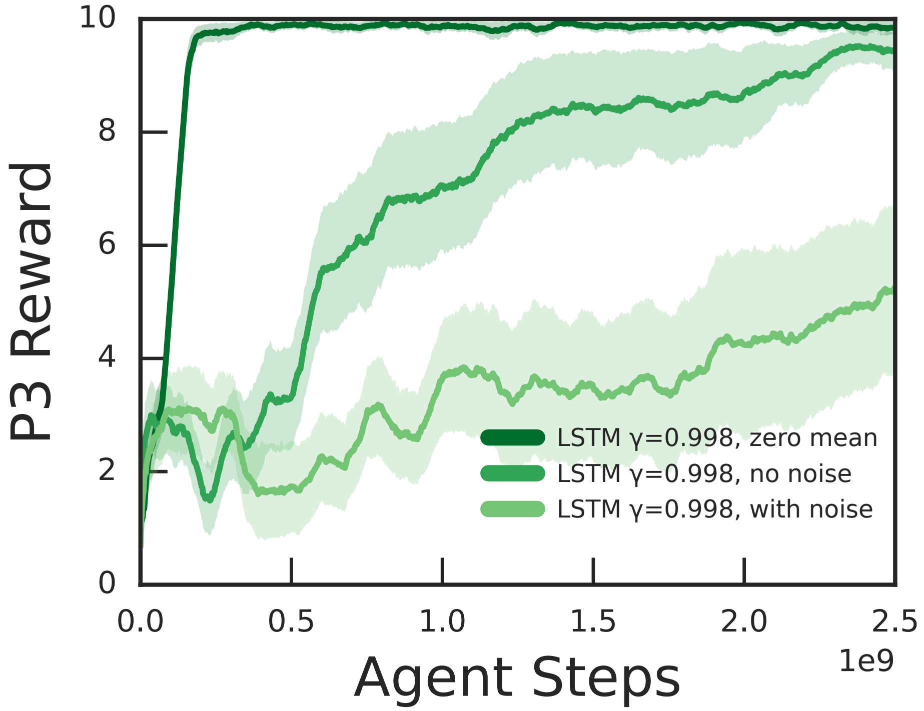

Supplementary Figure 11: **Constant vs. Variable P2 Reward.** The three curves shown are for the LSTM agent with $\gamma = 0.998$ in three variants of Key-to-Door: (i) zero apple reward, (ii) fixed number of apples each with reward 5, and (iii) the full level, which has a variable number of apples per episode but the same expected return as the fixed number of apples case. This analysis is discussed in Section 7. Variable P2 reward was maximally detrimental to performance.

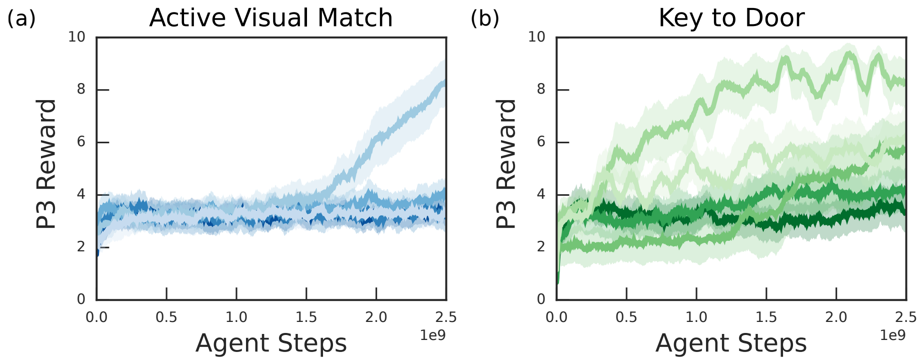

Supplementary Figure 12: **Learning Rate Search on Comparison Models ($\gamma = 0.998$).** Learning rates used were 3.2×10^{-7}, 8×10^{-7}, 2×10^{-6}, 5×10^{-6}, 1.25×10^{-5}, and are displayed from lightest to darkest in that order. In all analyses, the default learning rate of 5×10^{-6} performed best. *a.* RMA with $\gamma = 0.998$ on Active Visual Match with apple reward $r_{\text{ap[le}} = 1$. *b.* LSTM with $\gamma = 0.998$ on Key-to-Door task with variable apple reward as in Figure 4c in the main text, with P2 reward variance of 361.

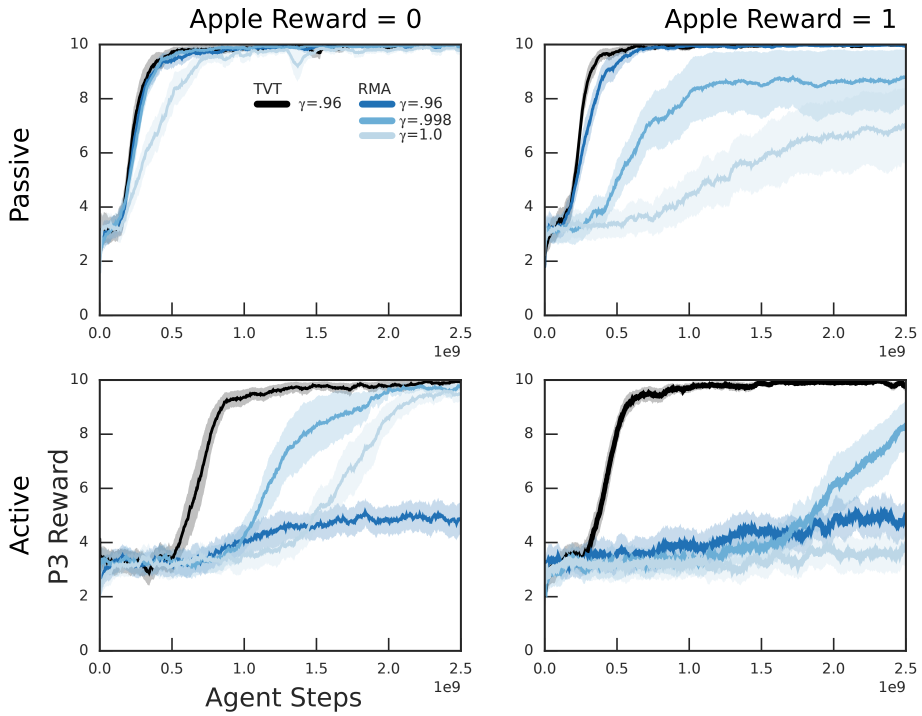

Supplementary Figure 13: **Effect of P2 Apple Reward in Passive and Active Visual Match Task.** *Upper Row.* On Passive, the RMA performed worse with larger discount factors, which are not needed to solve the task. *Lower Row.* On Active, the RMA models' performance at acquiring the distal reward degraded with the introduction of P2 reward. TVT remained stable with the introduction of P2 distractor reward.

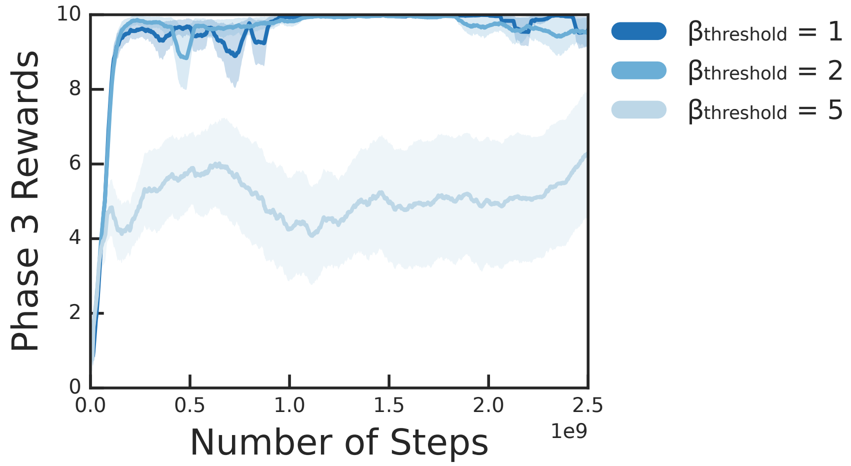

Supplementary Figure 14: **Effect of Varying Threshold to Activate Temporal Value Transport in Key-to-Door.** We saw no performance decline for a threshold value of 1, though performance deteriorated for $\beta_{\text{threshold}} = 5$.

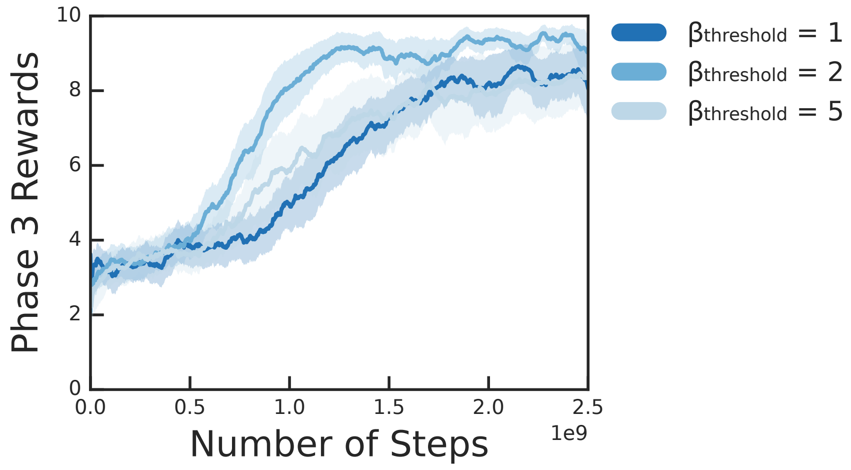

Supplementary Figure 15: **Effect of Varying Threshold to Activate Temporal Value Transport in Active Visual Match.** The performance of TVT was not greatly affected by the value of the threshold.

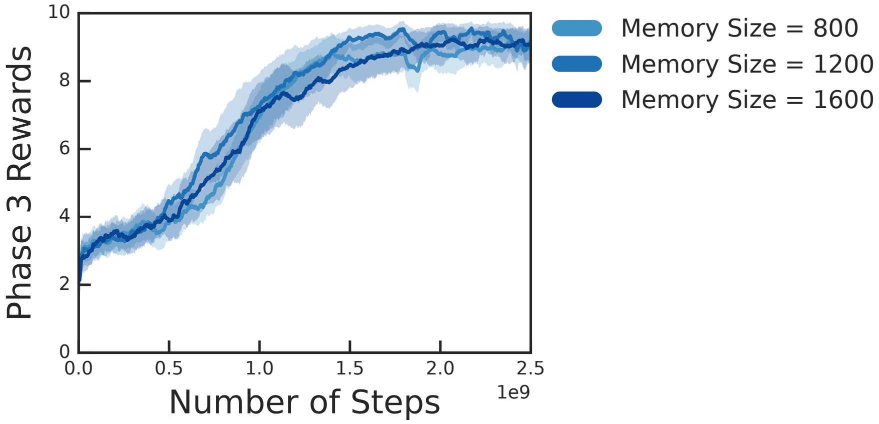

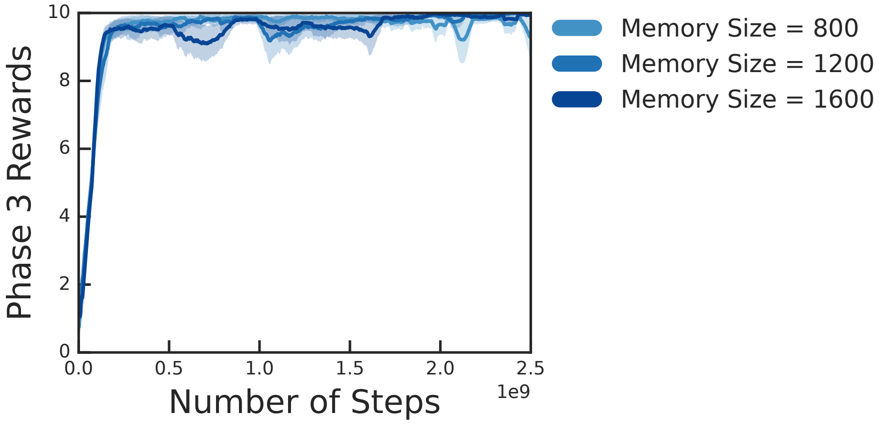

Supplementary Figure 16: **Comparison of Learning with Different Memory Sizes.** The memory system supports larger allocations of memory than needed for the level. *Top.* Performance on Key-to-Door. *Bottom.* Performance on Active Visual Match.

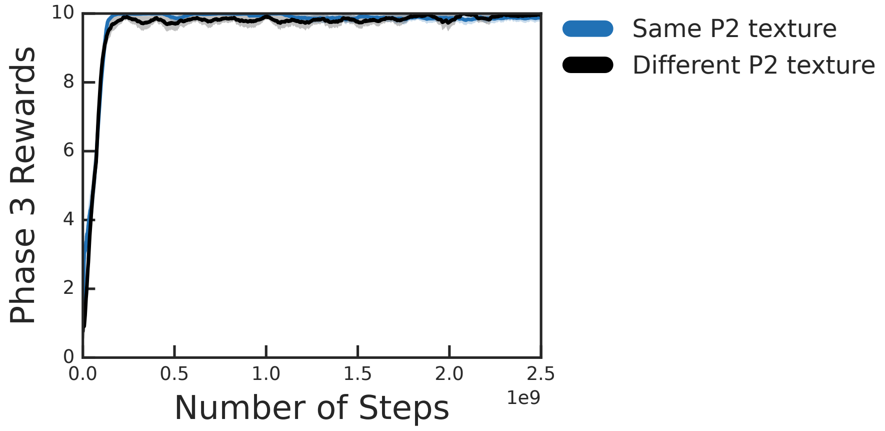

Supplementary Figure 17: **Key-to-Door Task: Robustness of TVT to Surface Similarities.** When the environment wall texture in phase 2 was made the same as the wall texture in phase 1, TVT still learned in the Key-to-Door task.

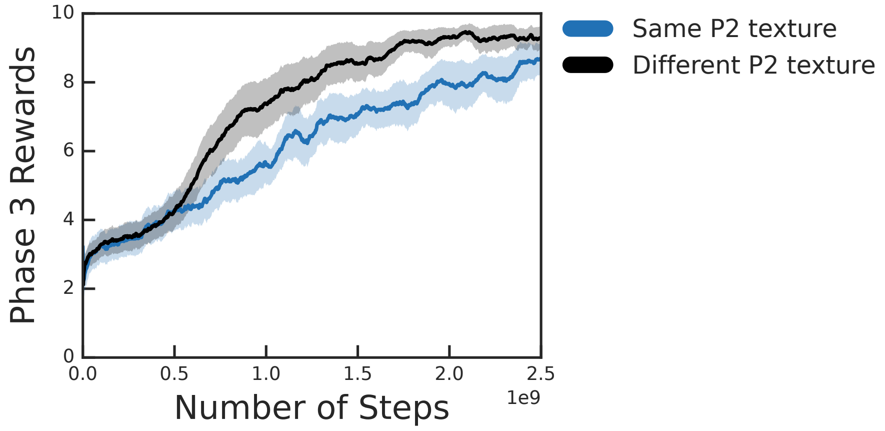

Supplementary Figure 18: **Active Visual Match Task: Robustness of TVT to Surface Similarities.** When the environment wall texture in phase 2 was made the same as the wall texture in phase 1, TVT still learned in the Active Visual Match task. There was a slight decrease in the speed of task acquisition.

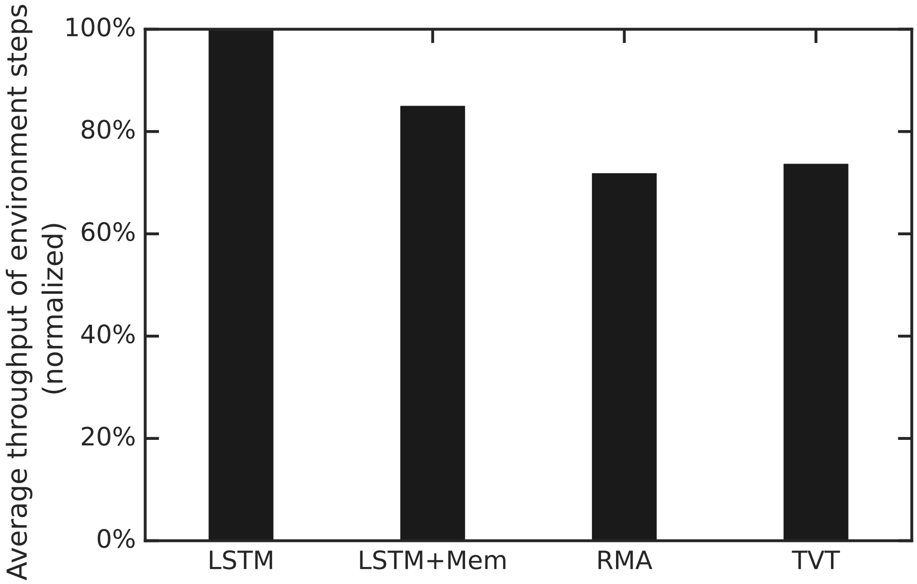

Supplementary Figure 19: **Speed of Different Models.** Although there is overhead to running the TVT memory system and algorithm, its throughput in processing environment steps is of the same order as a simple LSTM agent, left, LSTM with an external memory, and the RMA agent.

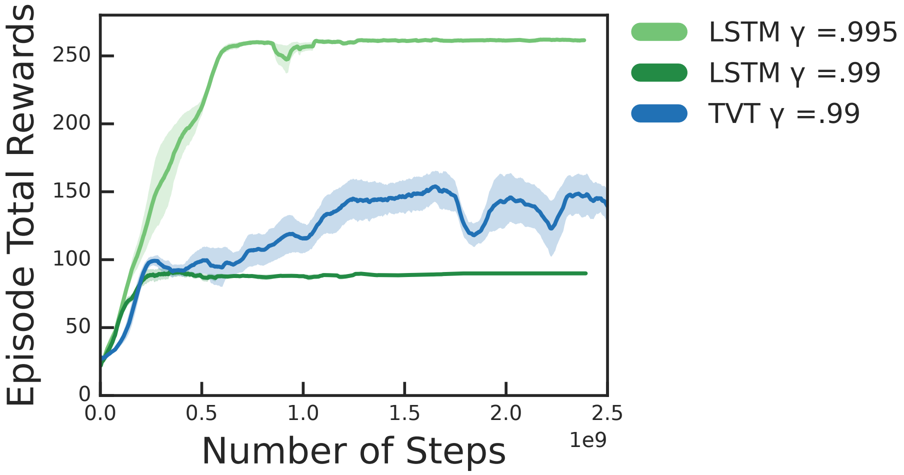

Supplementary Figure 20: **Learning Curves in Atari Bowling.** Using a higher discount factor than 0.99 (0.995) was sufficient for an LSTM agent to achieve state-of-the-art scores. TVT improved over the performance of the LSTM agent with the same discount factor of 0.99.

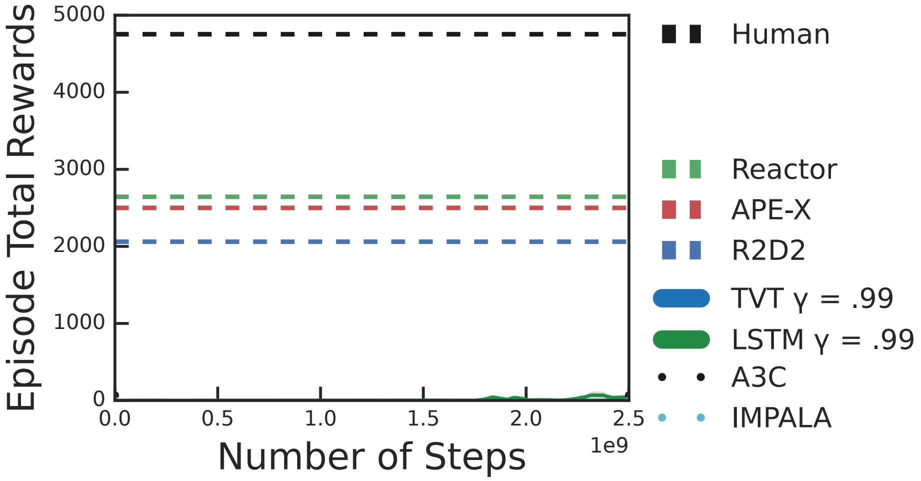

Supplementary Figure 21: **Learning Curves in Montezuma's Revenge.** We plot the learning curves of TVT and our control LSTM agent and show the final score of other previous published results. Numbers are extracted from Table 4 in [6] and from the original A3C [7]. The methods that worked best on this domain used off-policy experience replay; such methods make repeated learning updates from single experiences. The policy gradient methods without replay (including TVT, A3C, and IMPALA) performed worse than the replay-based Q learning methods (Reactor, APE-X, and R2D2), and all were below the human reported baselines. The very low probability of successfully encountering any reward in this game, alongside deterministic transition dynamics, implies that an effective strategy is to repeat any action trajectory that led to non-zero reward. In this regime, with very low probabilities of securing reward, TVT did not improve results.

Supplementary References

[1] Roberts, J. W. & Tedrake, R. Signal-to-noise ratio analysis of policy gradient algorithms. In *Advances in neural information processing systems*, 1361–1368 (2009).

[2] Beattie, C. *et al.* DeepMind Lab. *arXiv preprint arXiv:1612.03801* (2016).

[3] Leibo, J. Z. *et al.* Psychlab: a psychology laboratory for deep reinforcement learning agents. *arXiv preprint arXiv:1801.08116* (2018).

[4] Brady, T. F., Konkle, T., Alvarez, G. A. & Oliva, A. Visual long-term memory has a massive storage capacity for object details. *Proceedings of the National Academy of Sciences* **105**, 14325–14329 (2008).

[5] Krizhevsky, A., Nair, V. & Hinton, G. The CIFAR-10 dataset. *online: http://www.cs.toronto.edu/kriz/cifar. html* (2014).

[6] Steven Kapturowski, W. D. J. Q., Georg Ostrovski & Munos, R. Recurrent experience replay in distributed reinforcement learning. In *International Conference on Learning Representations* (2019).

[7] Mnih, V. *et al.* Asynchronous methods for deep reinforcement learning. In *International Conference on Machine Learning*, 1928–1937 (2016).
