# Supplementary material for: Optimizing agent behavior over long time scales by transporting value
Source: Nat Commun. 2019 Nov 19;10:5223. doi: 10.1038/s41467-019-13073-w (PMC6864102; doi:10.1038/s41467-019-13073-w)
Supplement: Supplementary file 3 — Description of Additional Supplementary Files [file 41467_2019_13073_MOESM3_ESM.pdf]

Description of Additional Supplementary Files

File Name: Supplementary Movie 1
Description: The Active Visual Match Level. Upper Left Panel. The agent's first person view is shown. Upper Right Panel. The top-down view of the environment is shown. Middle Panel. The empirical return, which is the sum of future reward, discounted by the agent's discount factor, is shown in orange. The agent's value prediction is shown in blue. Bottom Panel. The scale of the attention weight for a single memory read process (y-axis) is plotted against the memory slots (x-axis), which correspond to one slot per time point in the episode. Bottom Panel, right. The agent's memory read strength, defining the gain of its attention to the focused slots, is shown on the far right, along with the threshold value that, when crossed, triggers a value transport event. Movie Description. In phase 1, the agent actively explores the environment to find the visual cue on the wall in a two room environment. The agent's value prediction (blue) is above the level of the empirical return because it predicts transported value from phase 3. Phase 2, abbreviated in duration for this movie, involves collecting apples. In phase 3, the agent chooses the ground pad in front of the same cue color it observed in phase 1. Its memory read goes above threshold (we flash the read strength and attention weights in read when this happens). This corresponds to a value transport event. The highlighted slots have value transported to their corresponding time points.

File Name: Supplementary Movie 2
Description: The Key-to-Door Level. Upper Left Panel. The agent's first person view is shown. Upper Right Panel. The top-down view of the environment is shown. Middle Panel. The empirical return, which is the sum of future reward, discounted by the agent's discount factor, is shown in orange. The agent's value prediction is shown in blue. Bottom Panel. The scale of the attention weight for a single memory read process (y-axis) is plotted against the memory slots (x-axis), which correspond to one slot per time point in the episode. Bottom Panel, right. The agent's memory read strength, defining the gain of its attention to the focused slots, is shown on the far right, along with the threshold value that, when crossed, triggers a value transport event. Movie Description. In phase 1, the agent finds the key that it will need to open a door in phase 3. On picking up the key, a sensory cue, defined by a black flash, indicates the key has been acquired. The agent's value prediction (blue) is above the level of the empirical return because it predicts transported value from phase 3. Phase 2, abbreviated in duration for this movie, involves collecting apples. In phase 3, the agent observes the door and is able to open it because it has the key from phase 1. Its memory read goes above threshold (we flash the read strength and attention weights in read when this happens). This corresponds to a value transport event. The highlighted slots have value transported to their corresponding time points.

File Name: Supplementary Movie 3
Description: The Key-to-Door-to-Match Level. Upper Left Panel. The agent's first person view is shown. Upper Right Panel. The top-down view of the environment is shown. Middle Panel. The empirical return, which is the sum of future reward, discounted by the agent's discount factor, is shown in orange. The agent's value prediction is shown in blue. Bottom Panel. The scale of the attention weight for a single memory read process (y-axis) is plotted against the memory slots (x-axis), which correspond to one slot per time point in the episode. Bottom Panel, right. The agent's memory read strength, defining the gain of its attention to the focused slots, is shown on the far right, along with the threshold value that, when crossed, triggers a value transport event. Movie Description. In phase 1, the agent finds the key that it will need to open a door in phase 3. On picking up the key, a sensory cue, defined by a black flash, indicates the key has been acquired. The agent's value prediction (blue) is above the level of the empirical return because it predicts transported value from phase 3. Phase 2 involves collecting apples. In phase 3, the agent observes the door and is able to open it because it has the key from phase 1. Its memory read goes above threshold (we

flash the read strength and attention weights in read when this happens). This corresponds to a value transport event. The highlighted slots have value transported to their corresponding time points. Upon opening the door, the agent observes an image color cue (which happens to be green). In phase 4, it collects apples. In phase 5, the agent is able to choose the ground pad corresponding to the cue it observed in phase 3. The memory read goes above threshold and transports value to phase 3. The level demonstrates that temporal value transport can be used to propagate value across multiple stages: the value propagated to phase 1 is only achieved in phase 5.

File Name: Supplementary Movie 4

Description: The Latent Information Acquisition Level. Upper Left Panel. The agent's first person view is shown. Upper Right Panel. The topdown view of the environment is shown. Middle Panel. The empirical return, which is the sum of future reward, discounted by the agent's discount factor, is shown in orange. The agent's value prediction is shown in blue. Bottom Panel. The scale of the attention weight for a single memory read process (y-axis) is plotted against the memory slots (x-axis), which correspond to one slot per time point in the episode. Bottom Panel, right. The agent's memory read strength, defining the gain of its attention to the focused slots, is shown on the far right, along with the threshold value that, when crossed, triggers a value transport event. Movie Description. In phase 1, the agent explores by touching each of three objects in the room. When an object is good (the spinning suitcase here), it observes a green flash (but no reward, good or bad). If an object is bad, it observes a red flash (still with no reward). Phase 2 involves collecting apples. In phase 3, the agent retrieves memories from phase 1 and triggers value transport. It then correctly chooses only the object associated to a green flash.
